# Magnetic control over the fundamental structure of atomic wires

**DOI:** 10.1038/s41467-022-31456-4

**Published:** 2022-07-15

**Authors:** Sudipto Chakrabarti, Ayelet Vilan, Gai Deutch, Annabelle Oz, Oded Hod, Juan E. Peralta, Oren Tal

**Affiliations:** 1 Department of Chemical and Biological Physics, Weizmann Institute of Science, Rehovot, 7610001 Israel; 2 School of Chemistry and The Sackler Center for Computational Molecular and Materials Science, Tel Aviv University, Tel Aviv, 6997801 Israel; 3 Department of Physics, Central Michigan University, Mt. Pleasant, MI 48859 USA

**Keywords:** Magnetic properties and materials, Structural properties, Molecular electronics, Electronic properties and materials, Chemical physics

## Abstract

When reducing the size of materials towards the nanoscale, magnetic properties can emerge due to structural variations. Here, we show the reverse effect, where the structure of nanomaterials is controlled by magnetic manipulations. Using the break-junction technique, we find that the interatomic distance in platinum atomic wires is shorter or longer by up to ∼20%, when a magnetic field is applied parallel or perpendicular to the wires during their formation, respectively. The magnetic field direction also affects the wire length, where longer (shorter) wires are formed under a parallel (perpendicular) field. Our experimental analysis, supported by calculations, indicates that the direction of the applied magnetic field promotes the formation of suspended atomic wires with a specific magnetization orientation associated with typical orbital characteristics, interatomic distance, and stability. A similar effect is found for various metal and metal-oxide atomic wires, demonstrating that magnetic fields can control the atomistic structure of different nanomaterials when applied during their formation stage.

## Abstract (web summary)

Magnetic effects can emerge due to structural variations when the size of materials is reduced towards the nanoscale. Here, Chakrabarti et al demonstrates the opposite effect, showing that the interatomic distance in atomic wires changes by up to 20% depending on the orientation of an applied magnetic field.

## Introduction

One of the fascinating aspects of nanostructures is the new magnetic effects that are often developed as the dimensions of materials are reduced to the nanoscale. For example, while bulk gold (Au) is non-magnetic, gold nanoparticles may exhibit a finite magnetization^1^, and covering copper (Cu) with a film of C_60_ molecules can promote a ferromagnetic ordering at the Cu surface^2^. Over the years, structural manipulations that take advantage of the peculiar characteristics of nanomaterials were harnessed to achieve magnetic effects^3–8^. An interesting question that arises is whether the inverse relation can be realized. Namely, can magnetic manipulations lead to novel structural effects in nanomaterials?

Magneto-structural effects, such as magnetostriction^9–11^ and piezomagnetism^12–14^ are manifested as changes in the dimensions of magnetic materials in response to an applied magnetic field. These effects have diverse scientific and technological implications, either as undesirable effects in accurate measurements and device operation, or as means to achieve mechanical action via magnetic stimuli^15–18^. Since they are not limited to a specific length-scale, such effects can be observed, in principle, in nanometer-sized materials. However, their magnitude is typically of the order of parts per million, and therefore it is extremely challenging to detected them in nanoscale structures. Given the unique structural properties of nanomaterials, including dominant surface effects, reduced number of bonds between atoms (low atomic coordination), and reduced dimensionality, it is natural to expect the emergence of peculiar magneto-structural effects that are not necessarily revealed in bulk materials. Such effects, if detected, can shed new light on the physics of nanoscale materials and pave novel ways to control their properties.

**Fig. 1:**
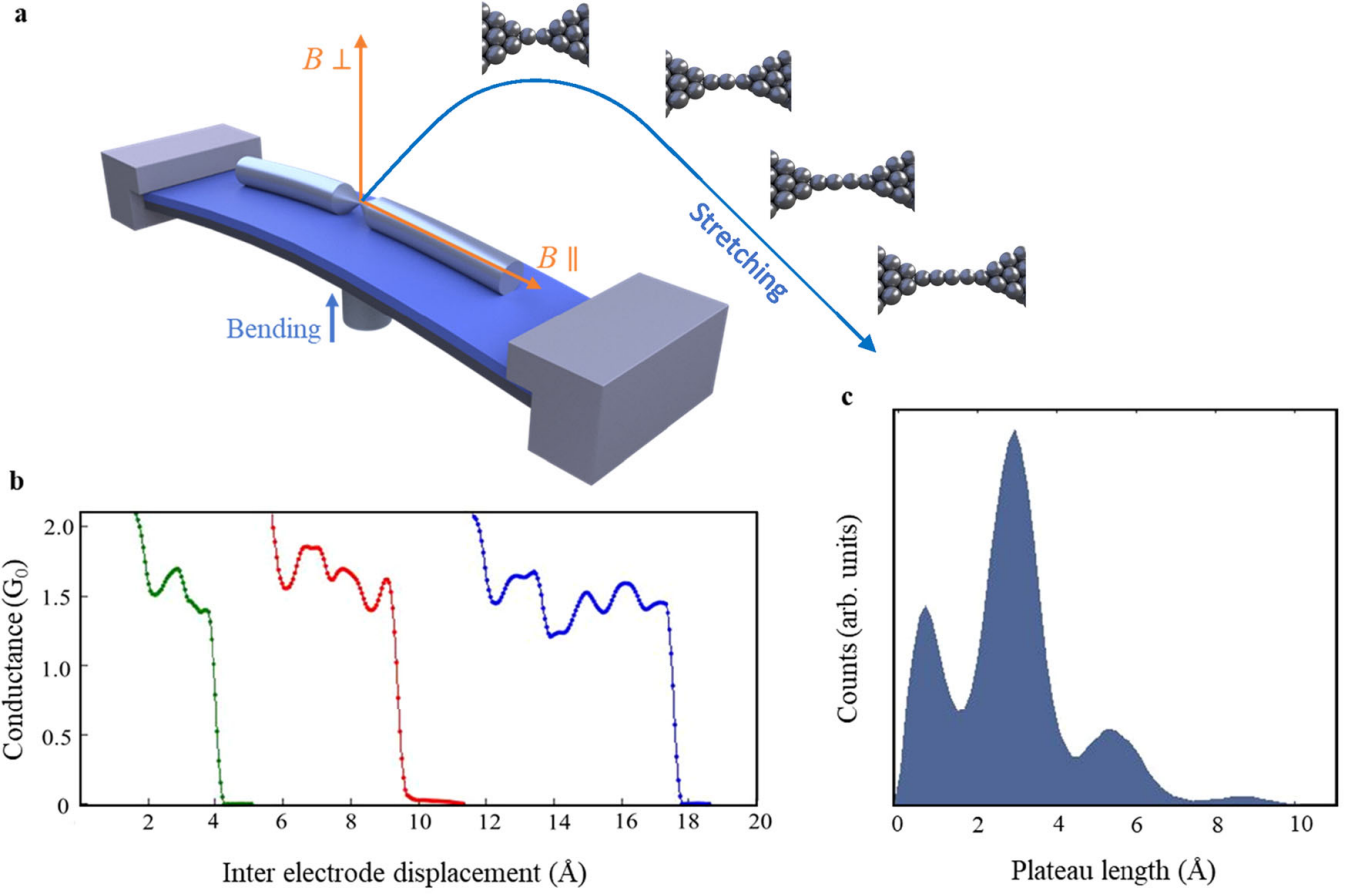
Formation of Pt atomic wires in a break-junction setup. **a** Illustration of a break-junction setup and sketches of stretched Pt atomic wires. When an atomic contact between the junction’s electrodes is stretched, atoms are pulled from the electrode to form an atomic wire. The process can be repeated by remaking and stretching the contact. A magnetic field can be applied parallel or perpendicular to the junction axis (orange arrows) during the formation of atomic wires. **b** Examples of conductance traces as a function of inter electrode displacement measured in the absence of a magnetic field and with a bias voltage of 20 mV. Right before complete junction breaking, the conductance drops to ~1.6 *G*_*0*_, the typical conductance of a constriction with a cross-section of one Pt atom between the Pt electrodes (see Supplementary Section 1). Further stretching yields a conductance plateau that ends when the junction breaks. **c** Length histogram composed of 10,000 such conductance traces that presents the number of times that a plateau with a given length was observed in the conductance range of 1.0–2.5 *G*_*0*_. The set of peaks is a signature for the formation of atomic wires with different lengths, and the inter-peak distance is a good measure of the interatomic distance^21,22,29,47,48^.

In the present study, using the mechanically controllable break-junction technique (Fig. 1a), we show that the magnitude and orientation of applied magnetic fields determine the structural properties of atomic wires during their formation. Specifically, we find that, when platinum (Pt) atomic wires are formed in the presence of an applied magnetic field parallel to the wire’s axis, the interatomic distance along the wire is shorter by up to ~20%, reaching a saturation at a magnetic field of about 1.25 *T* (Tesla*)*. The same field direction also promotes the formation of longer atomic wires. The situation is reversed when a perpendicular magnetic field is applied, leading to a longer interatomic distance by up to ~20%, with saturation at a similar magnetic field strength of about 1.25 *T*, and the formation of shorter atomic wires. These observations are substantiated by analyzing thousands of atomic wires. A minimal model that considers Zeeman splitting and thermal energy, successfully describes our experimental observations. The model, supported by ab-initio calculations, suggests that the direction of an applied magnetic field promotes the formation of stretched atomic wires with a specific magnetization orientation associated with a characteristic interatomic distance and stability. The microscopic mechanism underling this behavior is analyzed via further calculations and experiments that identify the central role of spin-orbit coupling (SOC), and show that changes in the magnetization directions lead to variations in orbital occupation accompanied by variations in interatomic binding. Importantly, we further present a similar magneto-structural activity in various metal and metal-oxide atomic wires, suggesting that the discovered magneto-structural effect may emerge in other nanoscale systems. The peculiar nature and magnitude of the revealed effect indicate that the structural characteristics of nanomaterials indeed promote unusual magneto-structural effects that are not found in bulk materials. Our findings thus reveal a previously unknown magneto-structural effect, where magnetic field manipulations at the fabrication stage can dictate the interatomic bonding properties in nanoscale systems. This effect can therefore be used to shape the structural properties of nanomaterials.

Our choice of atomic wires (Fig. 1a) as a testbed for the study of magneto-structural effects in nanoscale materials stems from their structural, magnetic, and electronic properties^19–35^. Suspended wires with a diameter of a single atom can be fabricated from pure metals^19–22^, alloys^23,35^, oxides^24,31^ or metals decorated with molecules^25,27^, with a length of up to ~10 atoms^26^. These wires have been used extensively to study fundamental aspects of charge, spin, and heat transport at the nanoscale^22,29–32^. Here, we first focus on suspended Pt wires that can be fabricated in a break-junction setup or a scanning tunneling microscope by stretching an atomic scale contact between two Pt electrodes. During this process, atoms are pulled one by one from the electrodes to form an atomic wire that bridges the two electrodes^21,22,28,29^. Unlike bulk Pt, which is paramagnetic (yet close to being ferromagnetic according to the Stoner criterion^30,36–38^), Pt atomic wires have been computationally predicted to turn ferromagnetic upon streching^36–42^. In fact, an experimental indication for magnetic activity in Pt wires was given by measurements of a finite magnetoresistance in this system^30^. Several density functional theory (DFT) based calculations predicted a reduction in the stability of Pt wires when magnetic ordering is included in the calculation^36–40^. Nevertheless, magnetic field induced structural modifications in atomic wires have never been studied before by either theory or experiment. Historically, the study of magnetism in Pt, has been an active research field for several decades^43^ with a rich literature on calculations of magnetic effects in Pt (bulk and surface) contaminated by magnetic impurities (e.g., Refs. ^44–46^).

## Results

Our experimental setup (Fig. 1a) allows us to study ensemble properties of Pt atomic wires. To that end, we form thousands of atomic wires and characterize their structure in the presence of constant magnetic fields of different strengths and directions. The Pt atomic wires are fabricated in ultra-clean conditions and cryogenic vacuum (at 5.1 K) between two Pt electrode tips (Fig. 1a), and their conductance, *G* (i.e., current over voltage), is recorded as a function of inter-electrode displacement^29^. Figure 1b presents such conductance traces, shifted horizontally for clarity. Right before breaking of the contact between the two beam segments (the electrodes), the conductance drops to approximately 1.6 *G*_*0*_ - the typical conductance of a contact with a single Pt atom cross-section^21,22,29,47^. Increasing the inter-electrode distance can break this contact or pull another atom from one of the electrode apices to form a short atomic wire that bridges the electrodes^21,22,29,47^. Further stretching may elongate the wire whenever the applied force on the bridging atoms reaches a critical value, at which an additional atom is pulled into the wire (provided that the wire does not break during the process). As seen in Fig. 1b, the elongation process leads to longer conductance plateaus at ~1.6 *G*_*0*_ with fine features^22,29^. These plateaus end when the wire breaks and the conductance drops abruptly to the tunneling regime. By repeated stretching until wire rupture followed by squeezing the electrodes to reform the contact (thus preparing the system for the next stretching step), we can sequentially fabricate thousands of atomic wires with different number of atoms and study their structural characteristics in the presence of a magnetic field (see Methods and Supplementary section 2).

**Fig. 2:**
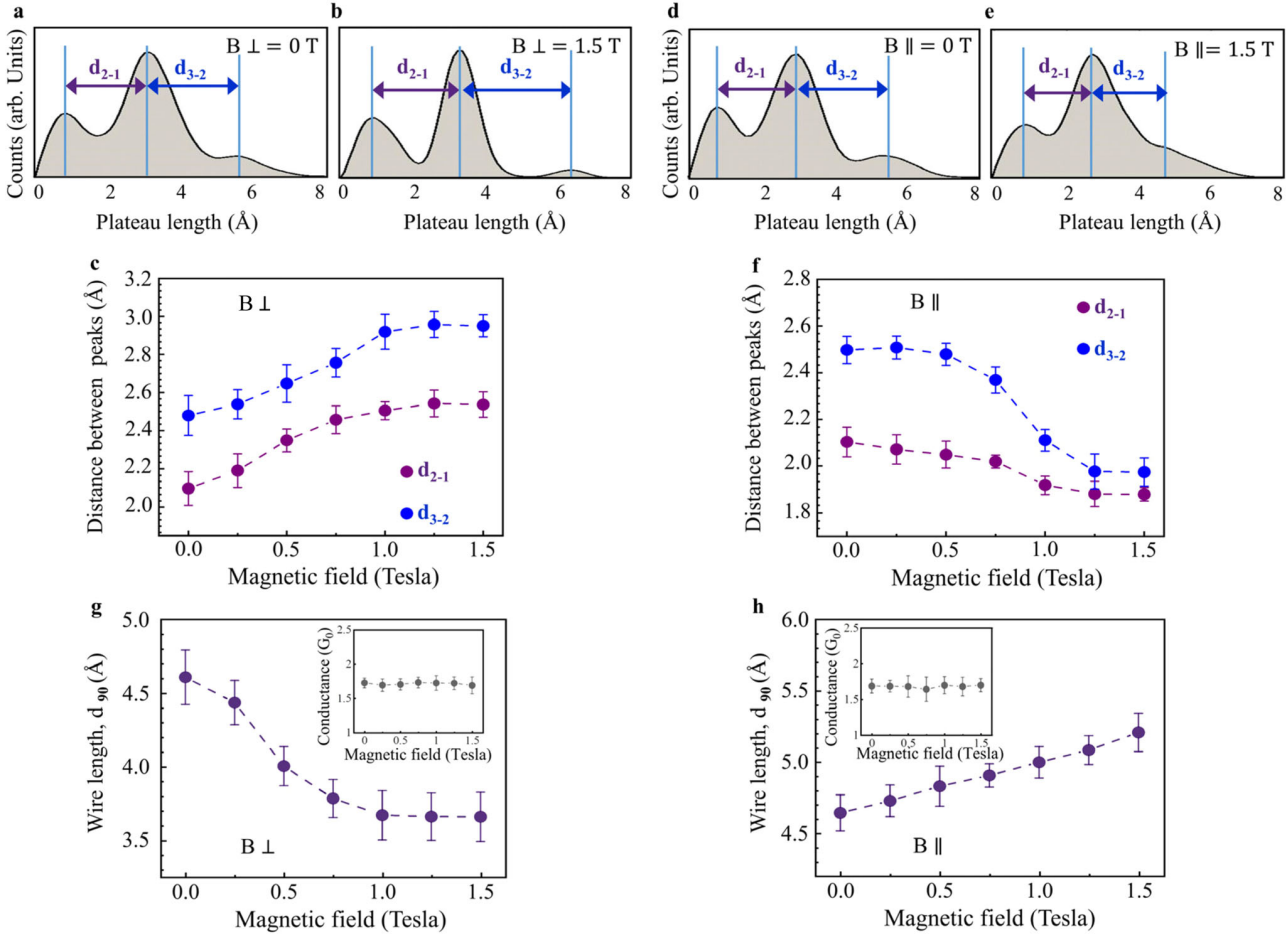
Magnetic-field-induced structural variations in Pt atomic wires. **a**, **b** Length histograms formed at zero applied magnetic field and at 1.5 *T* (Tesla) applied perpendicular to the junction’s axis, respectively, as defined in Fig. 1a. *d*_2-1_ and *d*_3-2_ are the inter-peak distances, which provide an indication for the average interatomic distance in the elongated wires. **c** Inter-peak distance as a function of perpendicular magnetic field. The overall increase in the inter-peak distance is 20.5 ± 0.6% for *d*_2-1_ and 18.9 ± 0.4% for *d*_3-2_. **d**, **e** Similar to (**a**, **b**), but with an applied magnetic field parallel to the junction’s axis, as defined in Fig. 1a. **f** Similar to c, but with a parallel magnetic field. Here, the overall reduction in the inter-peak distance as a function of parallel magnetic field is 10.5 ± 0.6% for *d*_2-1_ and 20.5 ± 0.6% for *d*_3-2_. **g**, **h** *d*_90_, a measure of the wire length, as a function of perpendicular (**g**) and parallel (**h**) magnetic field. Perpendicular (parallel) magnetic fields promote the formation of shorter (longer) wires. Insets: most probable conductance as a function of magnetic field perpendicular and parallel to the junction axis. See Fig. S1 for more details. Overall, fundamental structural properties of the Pt atomic wire are tuned by the direction and strength of the applied magnetic field. The data at each magnetic field in (**c**, **f**, **g**, **h**) is obtained from at least 8 length histograms that were collected during different experimental sessions. Each length histogram is based on 10,000 conductance traces measured under a bias voltage of 20 mV (similar analysis for 100 mV, and 180 mV shows no bias voltage effect, see Supplementary Section 3). The error bars provide the standard deviation of the averaged data.

Figure 1c presents a histogram of the length distribution of the 1.6 *G*_*0*_ conductance plateaus that are recorded during 10,000 repeated elongations of the Pt junction. This histogram is constructed by counting the number of times that a given ~1.6 *G*_*0*_ plateau length is recorded. The set of peaks in the length histogram seen in Fig. 1c indicates that atomic wires have a high probability to be elongated up to the length of the first peak and then break, or alternatively be elongated up to the length of the second peak and break, etc. This behavior is the fingerprint for the formation of atomic wires with different number of atoms^48^. The inter-peak distance can be considered as a measure of the average interatomic distance along the elongated wire^21,29,47^. For example, the distance between the third and second peaks, *d*_3-2_ (Fig. 2a) provides the average difference between the length of the wires associated with the second peak and wires longer by one atom. In the absence of an external magnetic field we find that *d*_3-2_ = 2.5 ± 0.2 Å and *d*_2-1_ = 2.1 ± 0.2 Å. The small difference between *d*_3-2_ and *d*_2-1_ is expected in view of previous DFT calculations that showed slightly different interatomic distances for atomic wires of different length^48,49^. The formation of Pt atomic wires that contain up to 6–7 atoms is experimentally observed but with low abundance^21,22^. Here, we choose to focus on the shortest (and more abundant) wires that reveal magneto-structural response. This allows for robust data analysis based on large ensembles.

Remarkably, Fig. 2a–c show that the inter-peak distance increases by up to 20.5 ± 0.6% in response to magnetic field perpendicular to the junction, saturating at a field intensity of about 1.25 *T*. In contrast, Fig. 2d–f reveal that the application of a parallel magnetic field leads to an inter-peak distance reduction of 20.5 ± 0.6% at the most, saturating at a similar magnetic field intensity. These observations indicate a significantly longer interatomic distance along the atomic wires when they are elongated in the presence of a perpendicular magnetic field, and a substantially shorter interatomic distance when a parallel magnetic field is applied during the formation of the wires. Notably, we did not detect any similar magneto-structural response in Au atomic wires (see Supplementary section 4). The insets of Fig. 2g, h, indicate that the most probable conductance of ~1.6 *G*_0_ is not affected by applied magnetic fields beyond the error bars uncertainty, though subtle conductance variations in the range of ±0.035 *G*_0_ are found (see Supplementary Section 1).

Next, we examine how the magnitude and orientation of the applied magnetic fields affect the overall length of the formed wires. To that end, we define the length *d*_*90*_ as a robust ensemble measure of the formed wire length, such that 90% of the formed wires are shorter than its value (see Supplementary Sections 5 and 6 for an analysis of different methods for wire length evaluation). Figure 2g, h show that a perpendicular magnetic field reduces *d*_*90*_, whereas a parallel magnetic field increases it (see Supplementary Section 7 for the magnetic effect on the number of atoms in the wires). These trends, which are opposite to the interatomic distance dependence on magnetic field, indicate that a perpendicular (parallel) magnetic field increases (decreases) the probability of wire rupture. This, in turn, hints that application of magnetic fields during wire formation influences the relative stability of the elongated wires such that longer (shorter) interatomic distances are correlated with lower (higher) wire endurance towards stretching and thus smaller (larger) overall wire length.

**Fig. 3:**
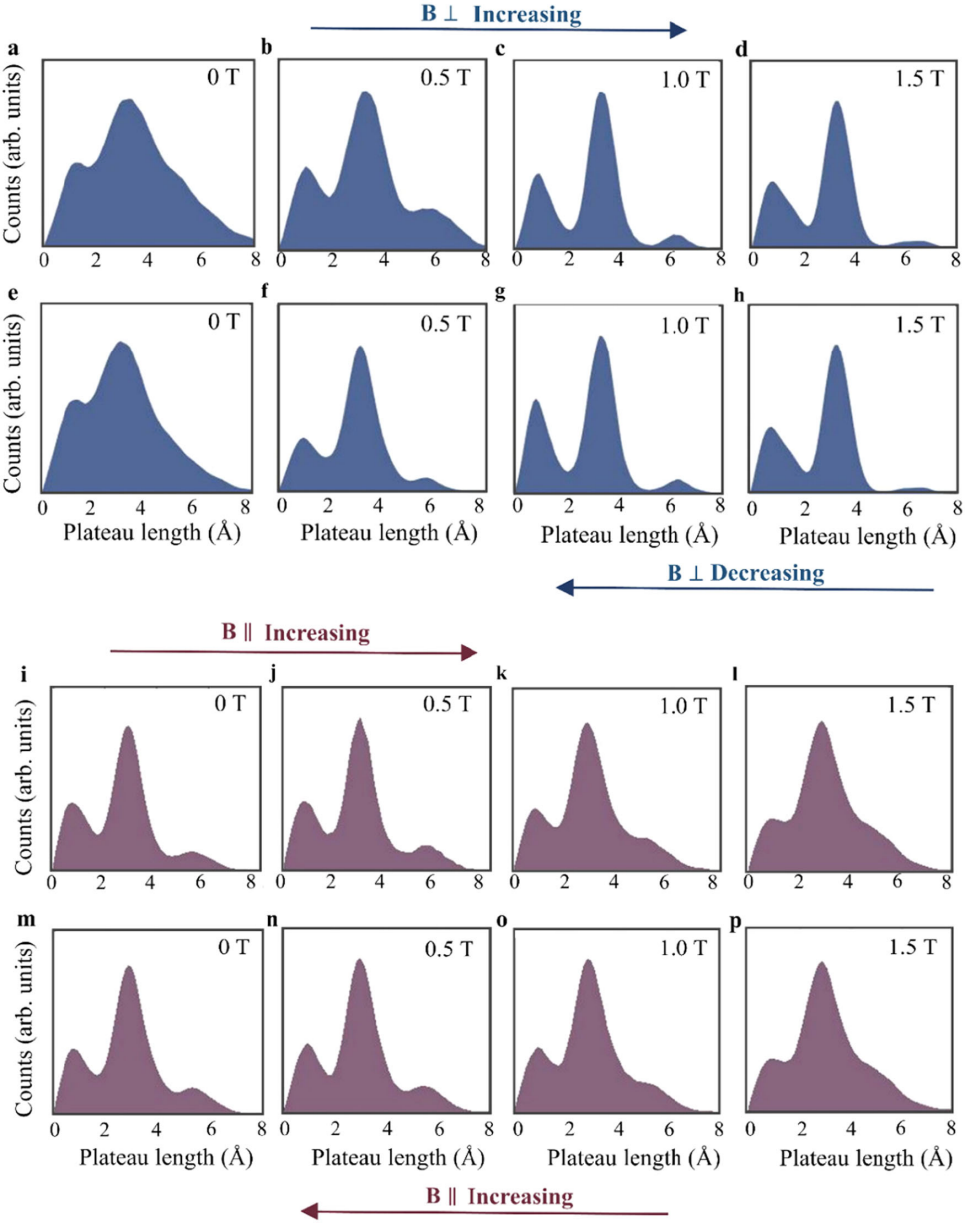
Magnetic field effect on the less-stable configurations of Pt atomic wires. **a**–**h** Length histograms based on 10,000 traces recorded consequently as a function of increasing (**a**–**d**) and decreasing (**e**–**h**) magnetic field perpendicular to the junction axis. The peaks’ sharpness reversibly increases with the applied magnetic field strength. **i**–**p** Similar measurements as a function of parallel magnetic field. In contrast to the former case, here the peaks’ sharpness decreases reversibly with increasing magnetic field strength. Length histograms with varied sharpness can be obtained at zero magnetic field by using different samples. We deliberately chose histograms with different sharpness at zero magnetic field to clearly present the (de)sharpening effect. A complementary analysis is presented in Supplementary Section 8, based on histograms of similar zero-field sharpness, allowing for a convenient quantitative comparison. We note that a similar analysis of Au wires control measurements (Supplementary Section 4) reveals no response to parallel magnetic field, whereas a slight increase in the peaks’ width is found for a perpendicular field. This effect will be further analyzed elsewhere.

Figure 3 reveals another aspect of the interplay between magnetic field and the structural properties of Pt atomic wires. The application of a perpendicular magnetic field (Fig. 3a–h) gives rise to sharper and better resolved peaks in the length histograms, whereas, in the presence of magnetic field parallel to the wires (Fig. 3i–p), wider peaks and relatively blur length histograms are obtained (see detailed analysis in Supplementary Section 8). To explain this, we note that the most frequently formed wires, and hence the most stable ones, tend to break at typical lengths given by the peak centers. In contrast, wires that break at length values between the peak centers are relatively rare and therefore considered to have a less-stable structure, or at least one unstable contact to the electrodes. Therefore, the size of the population of the less-stable configurations (off-peak counts) may serve as a sensitive probe for magnetically induced variations in wire perseverance during elongation. Namely, the peak sharpening (widening) observed with the application of a perpendicular (parallel) magnetic field indicates a reduction (increase) of the wire’s endurance during the formation process, in agreement with the analysis presented above (Fig. 2).

**Fig. 4:**
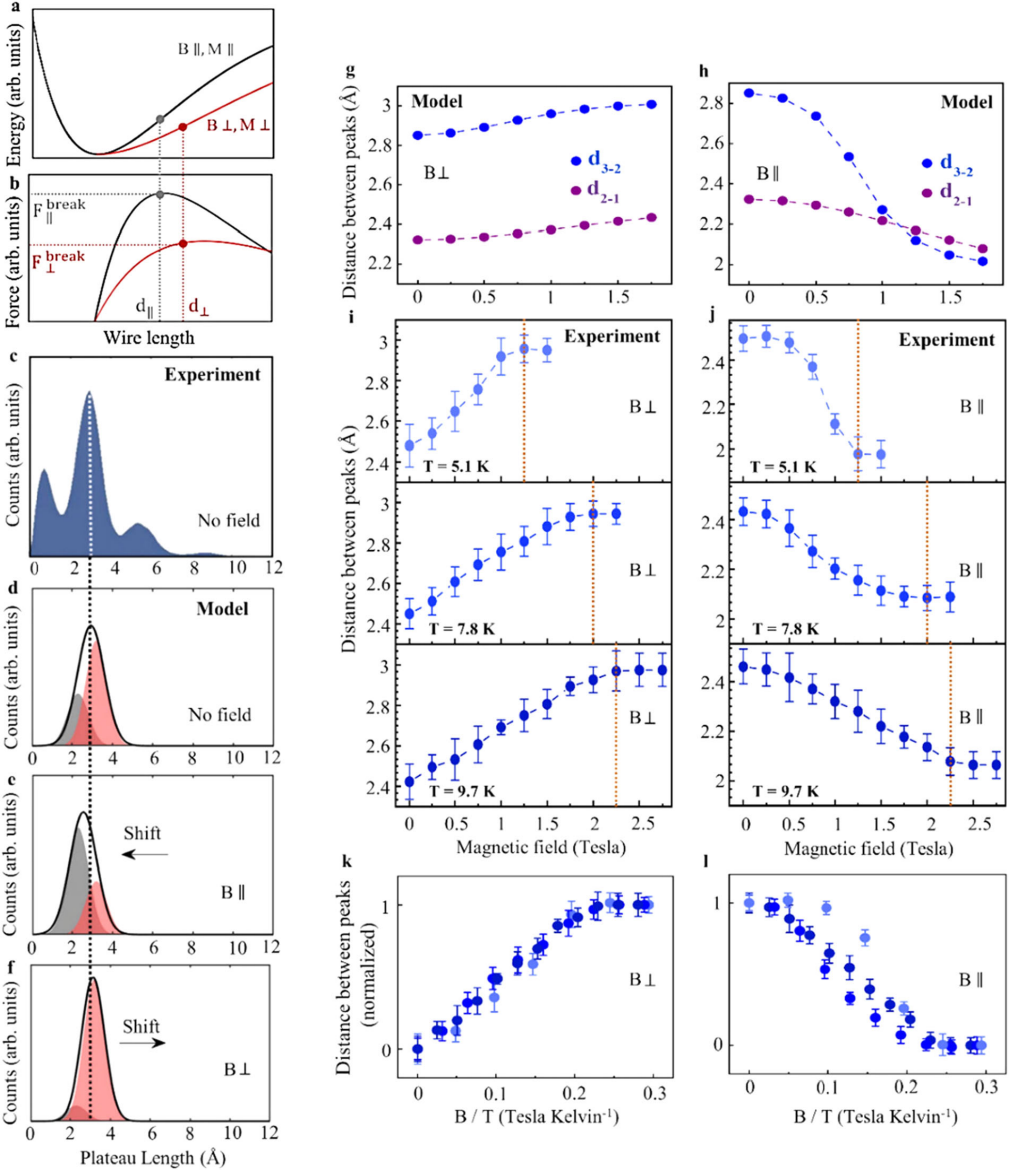
Minimal model and temperature dependent measurements. **a** Schematic curve of total energy as a function of wire length for parallel and perpendicular magnetic field and magnetization. **b** Same as (**a**) for the force. The breaking force (${{{{{{\rm{F}}}}}}}_{\parallel ,\perp }^{{{{{{\rm{break}}}}}}}$) is obtained at different wire length d_||,⊥_ for parallel and perpendicular magnetization and magnetic field orientations. **c** Experimental length histogram at zero magnetic field (same as Fig. 1c) presented for the sake of comparison to our model. **d**–**f** Illustration of the minimal model: (**d**) At zero magnetic field, two peaks represent the distribution of wire lengths with parallel (gray) and perpendicular (pink) magnetizations. In our model, these distributions are given by similar Gaussians multiplied by Zeeman-based Boltzmann weights that depend on the relative orientations and magnitudes of the magnetization and applied magnetic field. The distributions are centered at ${d}_{\parallel }^{n}$, ${d}_{\perp }^{n}$ for the *n*^th^ peak, and their summed contribution (black envelope) is adapted by setting the Gaussian widths to *σ* = 0.5 Å to fit the experimental peaks in (**c**). **e** At a finite parallel magnetic field, the two Boltzmann weights are different due to different Zeeman energy for parallel and perpendicular magnetizations. As a result, the heights of the two peaks are different, leading to a down-shift in the position of the maximum of the total distribution (black). **f** The two distributions at finite perpendicular magnetic field. Here, the different Boltzmann weights for parallel and perpendicular magnetizations lead to an up-shift in the position of the maximum of the total distribution (black). **g**, **h** Calculated *d*_2-1_ and *d*_3-2_ as a function of perpendicular (**g**) and parallel (**h**) magnetic field. ${d}_{\parallel }^{n}$, ${d}_{\perp }^{n}$ are determined by the onset of saturation in Fig. 2c, f. This model captures the opposite shifts in the inter-peak distance for parallel and perpendicular magnetic fields for both magnetization directions. **i**, **j** Inter-peak distance as a function of perpendicular (**i**) and parallel (**j**) magnetic field for different temperatures. **k**, **l** Same as in (**i**), (**j**), with magnetic field divided by the temperature.

To understand the observed magneto-structural effect, we present in Fig. 4a, b schematic curves of the wire’s energy as a function of elongation and its first derivative, the corresponding force (wire tension). The required force for breaking a wire is conventionally taken as the maximal force^40,42^, which corresponds to the inflection point in the energy curve. Our experimental data provide important information on the energy and force curves in the presence of applied magnetic fields. Figures 2c, f show that the distance between peaks is larger under a perpendicular magnetic field and smaller under a parallel field. Recall that the peaks indicate the wire length at which the wires tend to break, and the distance between peaks provides the average interatomic distance in the fully stretched wires^21,22^. Consequently, Fig. 2c, f reveal that the application of a perpendicular magnetic field promotes wire breaking at a longer interatomic distance, while applied parallel field leads to breaking at a shorter interatomic distance. In the force curves (Fig. 4b), this is manifested as a maximal force position located at a longer length (*d*_⊥_) for a perpendicular field and at a shorter length (*d*_||_) for a parallel field, with a similar influence on the inflection point of the energy curve (Fig. 4a). Figure 2g, h reveal that the formed wires are shorter when a perpendicular magnetic field is applied and longer when applying a parallel field. This implies a higher probability of reaching the breaking force under a perpendicular field before reaching the force needed to pull an atom from the electrodes, and vice versa for a parallel applied field. In the force curve (Fig. 4b), this is manifested as a lower (higher) maximal force when a perpendicular (parallel) magnetic field is applied, with corresponding variations in the energy curve (Fig. 4a).

Based on the experimental observations, we conclude that the application of parallel or perpendicular magnetic fields during wire fabrication leads to different binding energy characteristics. This can be rationalized in terms of magnetization alignment along the field direction, promoting the formation of wires with magnetization either parallel or perpendicular to their axis. The key point here is that atomic wires with parallel and perpendicular magnetizations have different total energy and force curves as illustrated in Fig. 4a, b. Our non-collinear spin DFT calculations (Supplementary Section 9) qualitatively support this picture by showing that the energy and force curves of stretched wires are indeed different for parallel and perpendicular wire magnetizations (see Fig. S19). This effect can be ascribed to the large spin-orbit interaction of Pt, as discussed below.

A puzzle remains, however, related to the fact that the Zeeman splitting energy associated with the experimentally applied magnetic fields is of the order of 10^−4^ eV, significantly smaller than the estimated energy difference between the parallel and perpendicular magnetization states in the stretched wire (Fig. S19). However, former observations indicate that after each insertion of an atom to the wire, the built tension and wire stiffness are considerably relaxed^47,50^. Consequentially, the interatomic distance approaches the equilibrium value. Under these conditions, the magnetization preference is suppressed^36–38,40–42^, thus facilitating the alignment of the magnetization along the magnetic field. If, upon stretching, an anisotropy barrier develops along with the increase in the energy splitting between parallel and perpendicular magnetization states (Supplementary Section 10), a lock-in of the magnetization orientation results. This picture thus explains how the spin orientation can be dictated by relatively small magnetic fields.

The following minimal model provides insights into the origin of the saturation observed in Fig. 2. Considering an ensemble of atomic wires, in the absence of an applied magnetic field, both wires with parallel and perpendicular magnetization configurations coexist at the experimental temperature of 5.1 *K*. According to this picture, each peak in the measured length histogram (Fig. 4c) is the sum of two distributions (black envelop in Fig. 4d), which represent the two ensembles of wires with perpendicular (pink; Fig. 4d) and parallel (grey; Fig. 4d) magnetizations. The former ensemble is twice as large as the latter since it consists of two perpendicular magnetization orientations versus one for the parallel orientation. For each peak, these two partially overlapping distributions (too close to be resolved experimentally) are represented by Gaussian functions centered at the breaking lengths for wires with parallel and perpendicular magnetizations (${d}_{\parallel }^{n}$ and ${d}_{\perp }^{n}$, respectively, for the *n*^*th*^ peak). The Gaussian widths are chosen as *σ* = 0.5 Å, such that their sum matches the experimentally observed histogram peaks in the absence of applied magnetic fields (Fig. 4c).

As discussed above, the application of parallel or perpendicular magnetic fields with respect to the wire’s axis alters these populations, favoring magnetization alignment along the field. This effect is accounted for by multiplying the Gaussian distributions by appropriate Boltzmann weights (see Supplementary Section 10). These weights consider the Zeeman energy due to the applied magnetic field parallel or perpendicular to the wire’s axis for the two magnetization orientations, as well as the competing thermal energy. We note in passing that apart from the parallel and perpendicular magnetization states other possible spin states, such as nearly anti-ferromagnetic configurations, have a considerably smaller Zeeman energy, and are hence neglected in our analysis. As illustrated in Fig. 4e, the application of a parallel magnetic field increases the wire population with a parallel magnetization (gray) and suppresses the population with a perpendicular magnetization (pink). This yields a shift in the envelope’s peak location (black) towards a shorter plateau length. The situation is inverted for a perpendicular magnetic field, as illustrated in Fig. 4f. According to this model, the shift in the peak location as a function of magnetic field asymptotically approaches saturation when the population of wires with magnetization aligned with the field dominates the peak’s locations. At zero temperature the locations of the peaks at saturation are ${d}_{\parallel }^{n}$ and ${d}_{\perp }^{n}$ for the corresponding field orientations. Therefore, as an input to our model, the locations of the peaks of the experimental length histogram at saturation are chosen as an approximation to ${d}_{\parallel }^{n}$ and ${d}_{\perp }^{n}$. Figure 4g, h present the model outcome for the inter-peak distance as a function of a parallel and perpendicular magnetic field strength. The observed shifts are similar to the experimental ones in Fig. 2c, f, with lower amplitudes. Varying the magnetization and Gaussian widths in a physically relevant range does not affect the qualitative results of the model (see Supplementary Section 11). Interestingly, a better agreement with the experimental shift amplitudes can be achieved by considering an average atomic magnetization larger than the value of 2.5 *μ*_*B*_ (*μ*_*B*_ - Bohr magneton) used here based on existing literature^36–42^.

According to this model, temperature increase should shift the onset of saturation to a higher magnetic field, since it is determined by a competition between the Zeeman and thermal energies. In contrast, the temperature effect on the distance between peaks at saturation should be negligible under our experimental conditions. To validate these model predictions, we present in Fig. 4i, j the experimentally obtained distance between peaks, *d*_3-2_, as a function of magnetic field, measured at different temperatures (see Supplementary Section 12). A clear upshift in the saturation field with temperature is indeed observed with no apparent variations in the amplitude of the distance between peaks beyond the experimental uncertainty. Figure 4k, l show that scaling the magnetic field by the temperature yields a similar saturation point for all six curves. These observations support the validity of our model and reveal the effect of moderate temperature variations on the magneto-structural response.

**Fig. 5:**
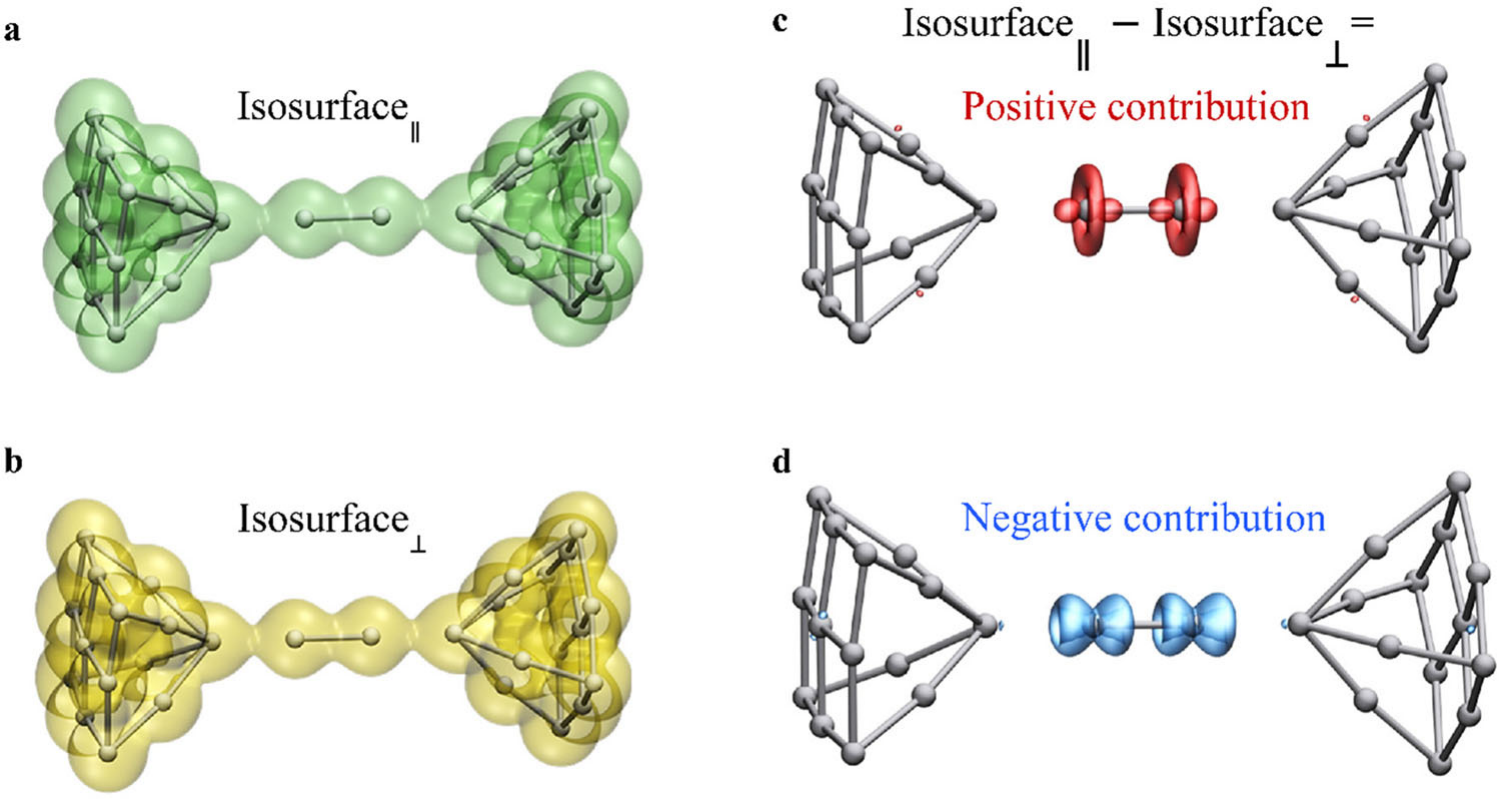
Calculated spatial distribution of the charge density for a short Pt atomic wire junction. **a**, **b** Isosurfaces of 1.6 Bohr^−3^ charge density for parallel (**a**; green) and perpendicular (**b**; yellow) magnetizations. **c**, **d** Isosurfaces of positive (**c**; red) and negative (**d**; blue) charge density differences between the parallel and perpendicular magnetizations isosurfaces. The positive and negative components in (**c**, **d**) reveal variations in the orbital contribution to the total charge density when changing the magnetization direction.

We now turn to examine the microscopic origin of the reported effect. Figure 5 shows calculated charge density isosurfaces for parallel (a, green) and perpendicular (b, yellow) magnetizations for a short Pt atomic wire junction, where no noticeable difference between the two is found at first sight. However, subtracting the charge density for the perpendicular magnetization case from that of the parallel magnetization clearly reveals the difference between the two. The positive component (c, red) can be seen as the added contribution to the charge isosurface when the magnetization changes from a perpendicular to parallel orientation and the negative component (d, blue) is the subtracted component due to the same change in magnetization orientation. The shape of the positive isosurface reveals a dominant contribution from ${d}_{{z}^{2}}$ (z is the wire axis), and the nodes in the negative isosurface indicates dominant contributions from *d*_*xz*_ and *d*_*yz*_ orbitals. We therefore find an enhanced contribution to the charge distribution of ${d}_{{z}^{2}}$ orbitals, on expense of *d*_*xz*_ and *d*_*yz*_ orbitals, when the magnetization is changed from perpendicular to parallel orientation. This is correlated with a larger Mayer bond order for the peripheral bonds prone to rupture in the stretched wire: 0.2124 for a parallel magnetization and 0.2084 for a perpendicular magnetization with a numerical error of ±10^−4^. The moderate change in the difference between bonding and antibonding states (given by the bond order) is accompanied by a significant difference in the binding force, as seen in Supplementary Fig. S18i. This indicates that near rupture, even mild changes in the bond order may translate to significant variations in the wire tension.

**Fig. 6:**
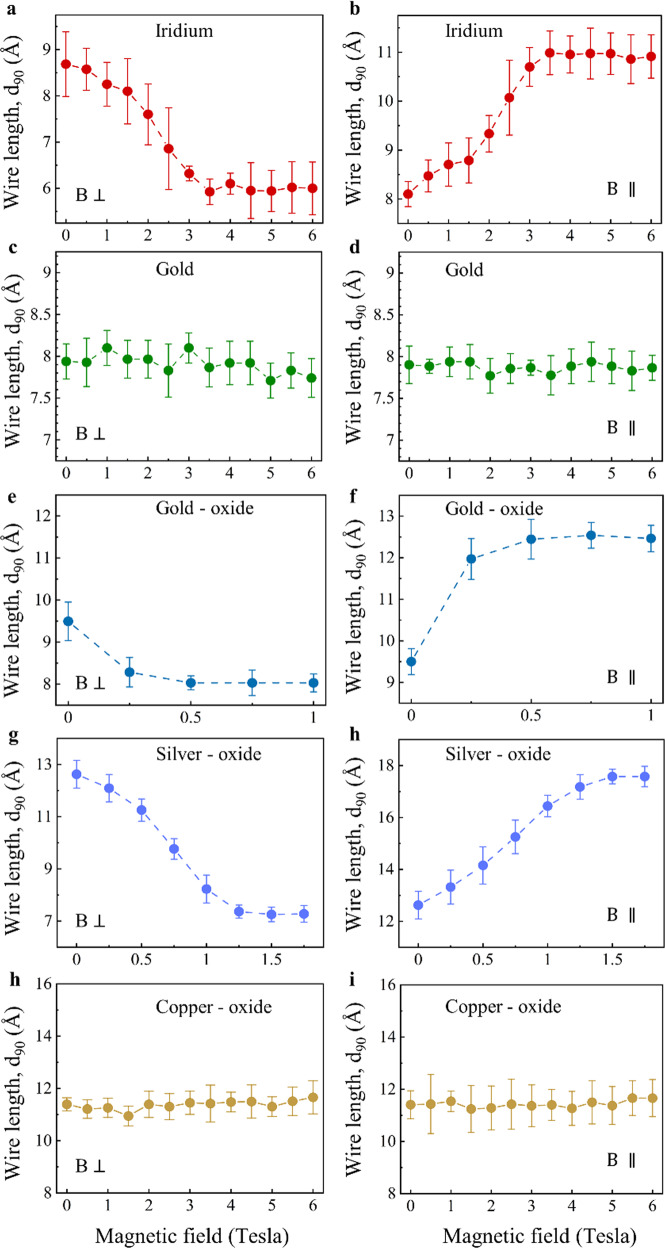
Magneto-structural effects in different atomic wires. **a**–**i** d_90_ as a function of perpendicular (left column) and parallel (right column) magnetic field for iridium (**a**, **b**), gold (**c**, **d**), gold-oxide (**e**, **f**), silver-oxide (**g**, **h**), and copper-oxide (**h**, **i**) atomic wires. Each data point provides the average *d*_90_ obtained from 5 different length histograms of 3000 traces each. Error bars present the standard deviation at each data point.

Figure 5 reveals that changes in the spin orientations lead to variations in the orbital characteristics. This is a clear signature of SOC, and an illustration of the link between magnetic-induced manipulations and the resulting structural changes. SOC is not explicitly considered in the above-mentioned phenomenological model but its role can be examined by a comparative experimental analysis, since the unveiled magneto-structural phenomenon is not limited to the case of Pt atomic wires. In fact, iridium (Ir) atomic wires show a similar magneto-structural response as that of Pt (Fig. 6a, b), yet saturating at ~3.5 *T* (compared to ~1.25 *T* for Pt). Considering the almost twice larger magnetic moment in Ir atomic wires than that of Pt wires^36,39^, one could expect a lower saturation magnetic field for Ir, in contrast to the experimental observations. We suggest that the larger SOC in Pt compared to Ir^51,52^ is the dominant factor that leads to a lower saturation field. In contrast, the lack of magneto-structural response for Au wires (Fig. 6c, d and Supplementary Section 4) indicates that only high SOC is not enough and the effect is not expected in the absence of magnetization, as in the case of Au wires^36,39^. To further test the role of SOC, we took advantage of the fact that Cu, Ag and Au have a different SOC (Cu<Ag< Au)^51,52^, and repeated our experiments with copper-oxide, silver-oxide, and gold-oxide (CuO, AgO, AuO) atomic wires that can be fabricated when the corresponding metal atomic contacts are exposed to oxygen^24^ (see Methods). Importantly, suspended CuO, AgO and AuO atomic wires are all expected to develop ferromagnetism with a comparable magnetization^53–57^. Figure 6e–h show no magneto-structural effect for CuO, and a finite effect for AgO and AuO. These observations indicate that a certain SOC magnitude is required in order to generate the probed magneto-structural effect, and for CuO the involved SOC magnitude is apparently below that threshold. The change in the wire length for AuO and AgO is comparable, but it saturates at different magnetic fields (~0.5 *T* and ~1.5 *T*, respectively). Since the expected magnetizations for AgO and AuO wires are comperable^53–56^ but the SOC of Au is higher, we find that (similar to the case of Pt and Ir) atomic wires with a higher SOC reveal higher structural sensitivity to magnetic field (i.e., lower saturation field). We note that the revealed magneto-structural response in AgO and AuO atomic wires is a first indication for magnetic activity in such atomic wires, calling for further study of these systems.

Despite the strong magneto-structural effect found in our experiments on Pt atomic wires, we could not find systematic variations in the most probable conductance of these wires as given by conductance histograms (Fig. 2g, h, Insets and Supplementary Section 1). However, this analysis has an uncertainty of ±0.1 *G*_0_, thus lower conductance variations cannot be detected. When elongating atomic wires, the conductance oscillates whenever an atom is added into the wire and the stretched linear wire is partially relaxed to a zigzag configuration. These oscillations, which have a typical range of ±0.05 *G*_0_, come from variations in the orbital overlap near the Fermi energy^29,48^. We find that the oscillation amplitude changes by about +0.035 *G*_0_ (−0.035 *G*_0_) on average in response to perpendicular (parallel) magnetic fields, (Supplementary Section 1). These observations provide a complementary indication for magnetic field induced variations in the orbital characteristics.

## Discussion

The discovered magneto-structural effect is fundamentally different from magnetostriction^9–11^ and piezomagnetism^12–14^. The latter phenomena are manifested as magnetic field induced variations in the shape of ferromagnetic and antiferromagnetic materials after their formation, due to changes in magnetization orientation. Moreover, these phenomena show hysteretic behavior. In the newly discovered effect, applying a magnetic field during wire formation promotes the fabrication of nanoscale architectures with a specific magnetization orientation and associated structural characteristics that are preserved in the suspended wire. Furthermore, in our measurements hysteresis is neither expected nor observed (Supplementary Section 13). To put the magnitude of the reported effect into context, the structural changes associated with magnetostriction and piezomagnetism are typically in the order of 10^−2^–10^−3^%, although larger magnetostriction of ~10^−1^% was found for specific rare earth transition metal alloys^58^. Minute length variations have also been reported in magnetic exchange force measurements on nickel-oxide substrates, where a height contrast of 0.5–1.5 picometer^59,60^ was at least partially associated with temporary structural relaxation in the scanning tip edge due to an exchange magnetic force, according to DFT calculations (e.g., Refs. ^61,62^). In contrast, the revealed magneto-structural effect is associated with orders of magnitude larger variations of up to ~20% in the interatomic distance (i.e., ~0.5 Å per interatomic bond).

To conclude, our observations provide a new perspective into the interplay between magnetism and nanoscale material structure, exemplifying that magnetic field direction and magnitude can control the structural properties of nanoscale materials during their formation. The demonstration of the studied magneto-structural effect for several systems, including Pt, Ir, AgO and AuO atomic wires, signifies its generality and indicates that it might be found in other nanoscale systems, possibly possessing sufficiently strong SOC and a sizable magnetization, such as metal and metal-oxide atomic-scale islands on surfaces and magnetically-active molecular junctions.

## Methods

### Experimental

The experiments are performed using a mechanical controllable break-junction set-up^19^ (Fig. 1a). The samples are composed of a notched Pt, Au, Ir Ag or Cu beam (Purity: 99.997%(Pt), 99.998%(Au), 99.9%(Ir), 99.997%(Ag), 99.9999%(Cu) 0.1 mm, 25 mm in length) attached to a flexible bendable substrate made of a 1-mm-thick phosphor-bronze plate covered by 100 *μm* insulating Kapton film. With the aid of a three-point bending mechanism including a piezoelectric element (PI P-882 PICMA), the substrate is bent to break the wire at its notch in cryogenic vacuum at a base temperature of 5.1 *K*. Breaking the beam forms a junction with a nanoscale gap between two ultra-clean atomically sharp apices that are used as electrodes. The two apices can be reconnected to have an atomic contact. Elongation of the contact in sub-Å resolution forms an atomic wire (metal-oxide wires are formed only after admitting oxygen into the contact), and the process can be repeated for thousands of times by reforming a multi-atomic contact with conductance of 50–70 *G*_0_ and elongating it to have another atomic wire. Oxygen (99.999%) is introduced to the cold sample via a heated capillary from an external reservoir^31^. The repeated elongation is performed at a rate of 20–40 *Hz*, while the conductance of the junction is measured simultaneously. The junction is biased with a d.c. voltage provided by a DAQ card (NI-PCI6221). The presented measurements are performed at a bias voltage of 20 mV. The resulting current is amplified by a current preamplifier (Femto amplifier DLPCA 200) and recorded by the DAQ card at a sampling rate of 50–200 *kHz*. The obtained current values are divided by the applied voltage values to extract the conductance. The inter electrode displacement is found by the exponential dependence of tunneling currents on the separation between the electrodes (see Supplementary Section 2). The magnetic field is applied using a vector superconducting magnet (horizontal ≤3T and vertical ≤9T) that provides a magnetic field parallel or perpendicular to the sample wire. The piezoelectric element that is used to bend the sample is driven by the same DAQ card connected to a piezo driver (Piezomechanik SVR 150/1). The measurements were obtained for five different samples for each metal and three different samples for each metal-oxide, using different cryogenic probes. A similar behavior was found for all measured samples of the same type. The error bars represent the variations between independent measurements, serving as a reliable measure for the degree of experimental uncertainty.

### Data analysis

Conductance histograms, length evaluation, and Gaussian fittings are done by in-house MatLab codes. The evaluations of the peak centers and widths in the length histograms are derived by deconvolution into Gaussian peaks using the ‘fit.m’ MatLab function with ‘gauss4’ fit type and the ‘NonlinearLeastSquares’ method. Percentile values (e.g., d_90_) are derived by the ‘prctile.m’ function.

### Calculations

The underlying assumptions of the minimal model were validated using fully unconstrained non-collinear spin DFT calculations including SOC^63–65^. All DFT calculations were performed using the Gaussian suite of programs^66^ using the PBEh hybrid density functional approximation^67–69^, which admixes 25% of orbital-dependent Hartree-Fock exchange with 75% of PBE exchange and 100% PBE correlation, and the Stuttgart-Cologne energy-consistent relativistic (10 electrons) small-core effective core potential, including the spin-orbit component, and the corresponding aug-cc-pVDZ-PP basis-set^70^. Our choice of hybrid functionals is based on their success in treating transition metal complexes and their use in the context of strongly correlated systems^71,72^. The details of these calculations and the corresponding model systems are provided in Supplementary Section 9.

## Supplementary information

Supplementary Information

## Data Availability

The data that support the findings of this study are available from the corresponding authors upon reasonable request due to the huge volume of the raw data collected in this study.

## Code availability

The non-commercial computer codes that support the findings of this study are available from the corresponding authors upon reasonable request.
