# Supplementary material for: Magnetic control over the fundamental structure of atomic wires
Source: Nat Commun. 2022 Jul 15;13:4113. doi: 10.1038/s41467-022-31456-4 (PMC9287401; doi:10.1038/s41467-022-31456-4)
Supplement: Supplementary file 1 — Supplementary Information [file 41467_2022_31456_MOESM1_ESM.pdf]

Supplementary Information

Magnetic control over the fundamental structure of atomic wires

Sudipto Chakrabarti[1], Ayelet Vilan[1], Gai Deutch[1], Annabelle Oz[2], Oded Hod[2], Juan E. Peralta[3] & Oren Tal[1]

[1] Department of Chemical and Biological Physics, Weizmann Institute of Science, Rehovot 7610001, Israel

[2] School of Chemistry and the Sackler Center for Computational Molecular and Materials Science, Tel Aviv University, Tel Aviv 6997801, Israel

[3] Department of Physics, Central Michigan University, Mt. Pleasant, Michigan 48859, United States

The supplementary information consists of 13 sections, as listed below:

Supplementary section 1: Conductance as a function of magnetic fields in Pt atomic wires

Supplementary section 2: Length calibration

Supplementary section 3: Applied voltage effect on the magneto-structural response of Pt wires

Supplementary section 4: Magneto-structural measurements in Au atomic wires

Supplementary section 5: d_{90} and its response to magnetic field

Supplementary section 6: Comparison between different wire length evaluations

Supplementary section 7: magnetic field effect on the number of atoms in Pt wires

Supplementary section 8: Magnetic field effect on the peak sharpness in the length histograms of Pt atomic wires

Supplementary section 9: First-principles calculations

Supplementary section 10: Description of the model

Supplementary section 11: Model sensitivity with respect to input parameters

Supplementary section 12: Temperature dependent measurements

Supplementary section 13: Absence of hysteresis

Supplementary section 1: Conductance as a function of magnetic fields in Pt atomic wires

Most probable conductance analysis:

We measure the conductance *G* (*G=1/R*, where *R* is the resistance) as a function of magnetic field by fixing the field and collecting 10,000 conductance traces as a function of inter-electrode displacement (see Fig. 1b of the main text). These traces are used to construct conductance histograms at different magnetic fields as seen in Figs. S1a-h. The observed peak at about 1.6 G_0 in these histograms provides the most probable conductance of the Pt atomic wires. The conductance above the 1.6 G_0 peak is the outcome of contacts with more than one atom in their cross section that are formed before wire elongation, and the tail at low conductance stems from tunneling conductance that follows wire rupture. The figures show a sequence of conductance histograms taken one after the other at different magnetic field magnitudes, either perpendicular or parallel to the junction's axis. We do not observe any indications for a systematic shift in the conductance peak at $\sim$1.6 G_0 as a function of magnetic field. Figs. S1i,j show the average value of the conductance peak maximum as a function of perpendicular (i) and parallel (j) magnetic fields for several measurement sets, similar to the two sets presented in Figs. S1a-h. Each data point is an average based on at least 8 different conductance histograms collected at a given magnetic field but in different experimental sessions. As exemplified in Figs. S1i,j , in the limit of the experimental error, we do not find any magnetic-induced variations in the conductance of Pt atomic wires.

We note that our analysis is insensitive to possible magnetoresistance variations that might exist in atypical and infrequently-formed structures of Pt atomic wires or to finite magnetoresistance with an arbitrary sign for different junction realizations, since we average-out their possible contributions. Furthermore, we cannot detect very small magnetic-induced conductance variations of the order of a few percent, since they are in the range of the minor differences in the peak locations sometimes detected in different conductance histograms regardless of the presence or absence of an external magnetic field. Such variations can possibly stem from changes in the atomic structure of the electrodes. These characteristics of ensemble-based analysis may explain the discrepancy between our transport measurements and the one reported in Ref. 1 (Ref. 14 in the main text).

The approach that we use in our analysis has an important advantage. In magnetoresistance measurements, variations in resistance (or conductance) can be an outcome of magnetostriction effects rather than pure magnetoresistance of electronic origin. Such undesirable magnetostrictive effects can take place when the inter-electrode distance is kept constant after an atomic wire is formed and then a magnetic field is swept. In this case, conductance variations as a function of magnetic field can stem from magnetic-induced structural variations, such as: (i) extrinsic magnetostriction-induced changes in the dimensions of the sample or sample-holder that lead to changes in the interelectrode distance; (ii) intrinsic magnetostriction, where the interelectrode distance remains intact but possible changes in the wire length and tension in response to magnetic field variations may lead to

conductance changes. In our measurements, we avoid these unwanted effects by performing repeated conductance measurements during wire elongation under a fixed magnetic field. As mentioned in the main text, minor conductance variations due to the reported magnetic effect on the interatomic distance are expected to be smaller than the uncertainty range of the presented ensemble analysis in Figs. S1i,j.

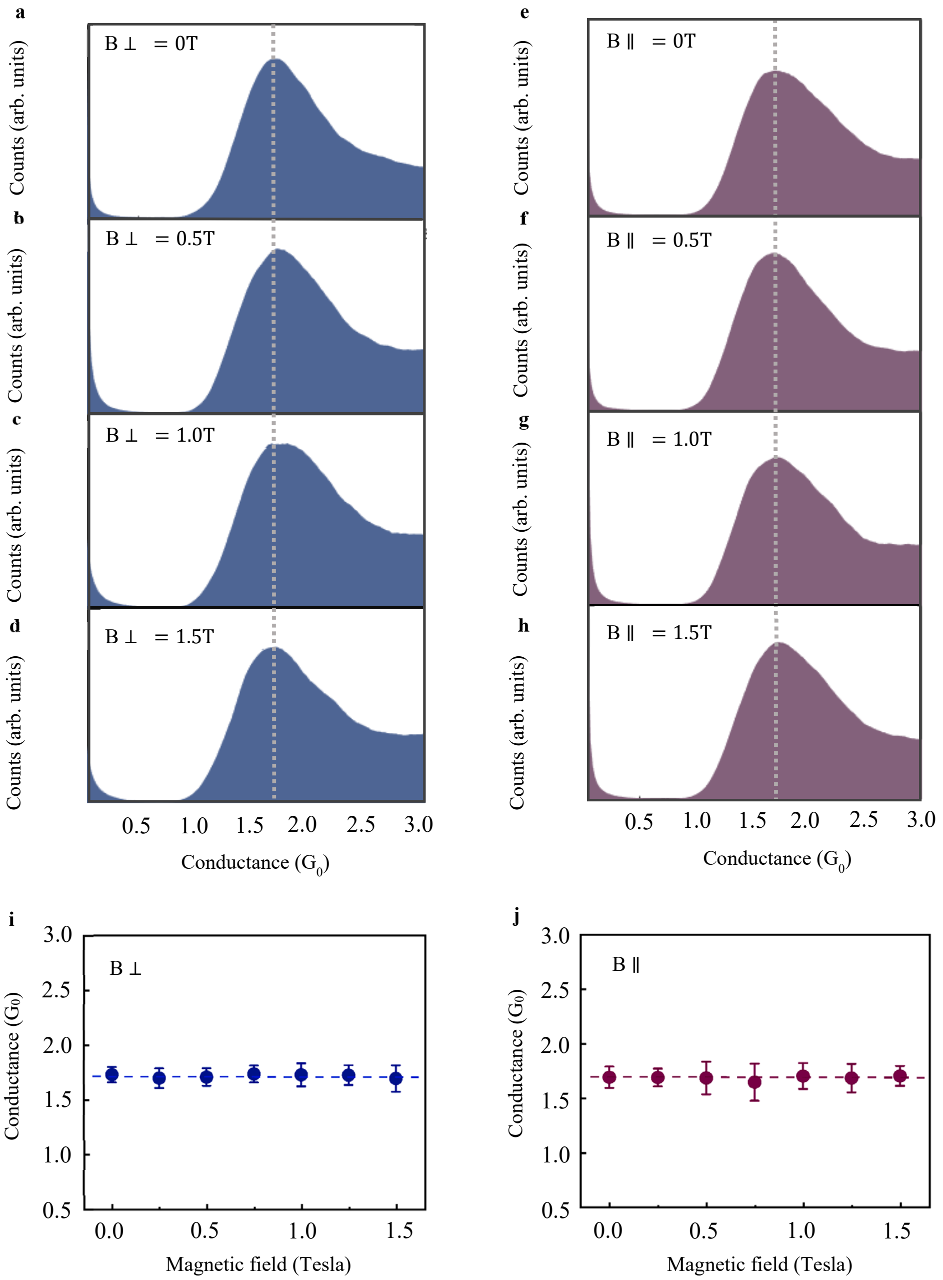

Fig. S1 Conductance as a function of applied magnetic fields during the formation of Pt atomic wires. a-d, Conductance histograms collected at different magnetic fields perpendicular to the junction's axis. **e-h,** Similar to a-d but for a parallel magnetic field. **i,** Conductance at the peak maximum as a function of magnetic field perpendicular to the junction's axis. **j,** Similar to i but for a parallel magnetic field. In i,j each data point is an average of the peak value, based on at least 8 different conductance histograms taken in different experimental sessions. Each such histogram is composed of 10,000 conductance traces, measured under a bias voltage of 20 mV. The error bars are the standard deviation of peaks' maxima. No magnetoresistance is detected within the measurement uncertainty. Figs. S1i,j appear in the main text as Insets in Fig. 1g,h.

Conductance oscillations during wire elongation:

As mentioned above and in the main text, we do not observe variations in the most probable conductance larger than several percent. However, we find clear indications for the effect of applied magnetic fields on electronic transport when focusing on conductance as a function of elongation. Figure S2, presents the average conductance measured during wire elongation under parallel (a), zero (b), and perpendicular (c) applied magnetic fields. Clear conductance oscillations are seen riding on a decaying conductance baseline. These oscillations are ascribed to variations in the local orbital structure during the elongation process of Pt atomic wires[2,3]. Specifically, a relaxed wire has a zigzag structure, however, when the wire is stretched it becomes linear. This results in an enhanced orbital hybridization and higher density of states at the Fermi energy, translated to conductance increase. Further stretching results in either rupture or the insertion of another atom into the wire, and partial relaxation of the wire back to the less-conducting zigzag configuration. Therefore, during wire elongation the conductance oscillates (Fig. S2) and the magnitude of these oscillations probes the extent of orbital overlap at the Fermi level[3].

Focusing on the influence of parallel and perpendicular magnetic fields on the amplitude of the conductance oscillations (Fig. S2), we find that a perpendicular magnetic field enhances the oscillations and a parallel field suppresses them. These findings provide another indications that magnetic fields affect the orbital structure, and specifically a perpendicular (parallel) magnetic field enhances (suppresses) orbital overlap at the Fermi level. Transport measurements probe the states located in a window around the Fermi energy (about 20 meV in our case). In contrast, the magneto-structural response probes variations in the orbitals that are involved in the interatomic bonds that are not limited to the vicinity of the Fermi level. Thus, the two types of analysis complement each other. We note that the magnetic induced changes in the amplitude of the oscillations is clear, though it is limited to a few present and therefore not seen in the most probable conductance analysis.

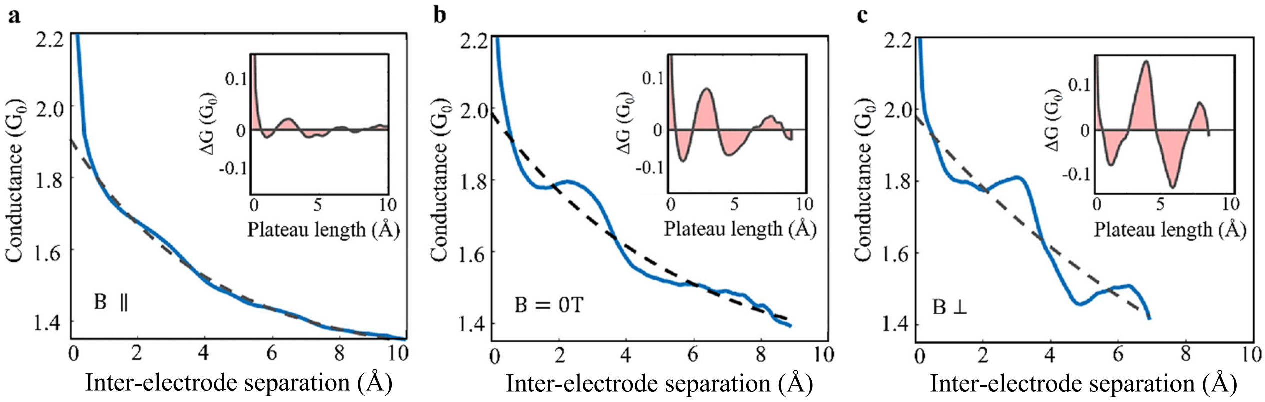

Fig. S2 Magnetic field effect on the average conductance traces of Pt atomic wires. a-c, Average conductance (blue) as a function of inter-electrode separation under a parallel magnetic field of 1.5 T (a), no applied magnetic field (b), a perpendicular magnetic field of 1.5 T (c) for applied bias voltage of 20 mV. The dashed black line is an exponential fit. Insets, difference between the average traces and the exponential fit. The observed conductance oscillations are enhanced (suppressed) when a perpendicular (parallel) magnetic field is applied. The data of each panel is based on 10,000 conductance traces taken at an applied bias voltage of 20 mV.

Supplementary section 2: Length calibration

The relative displacement of the electrodes in our break junction setup is proportional to the voltage applied on the piezo element (piezo voltage). We find the calibration constant between the piezo voltage and the inter-electrodes displacement based on the exponential decay of the conductance with distance in the tunneling regime (Fig. S3) as described in more details in Ref. 4 and its supplementary materials. Specifically, we collect histograms of the calibration constant based on thousands of traces of conductance as a function of piezo voltage to obtain a statistically reliable calibration (Fig. S3, Inset). The measured average inter-peak distance at zero applied magnetic field, which is determined based on the found calibration constant is in agreement with the value of 2.3$\pm$0.2 Å that appears in the literature[5,6]. We note in passing that in our work the key information is the magnetic-field-induced variations in the inter-peak distance and the d_{90} rather than their absolute values.

The calibration factor may, in principle, vary in response to different magnetic fields. This is due to magnetostriction response that can affect the dimensions of the sample and sample-related components (the phosphor-bronze sample substrate, sample holder, etc.). Therefore, the relevant setup components and the sample itself are composed of materials with negligible magnetostriction. We verify that magnetic fields do not affect the calibration factor by repeating our measurements with gold (Au) atomic wires, which are not expected to exhibit any magnetic response in the relevant magnetic field range. As can be seen in Supplementary section 3, we did not detect any variations in the interatomic distances and d_{90} in response to magnetic fields for the case of Au atomic wires. This indicates that the calibration factor in our experimental setup is not affected by magnetic fields, for any practical purpose.

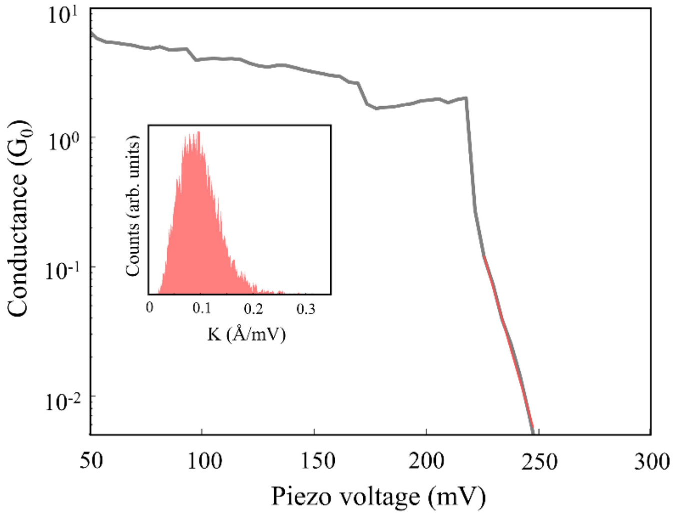

Fig. S3 Length calibration by conductance dependence on piezo voltage. Conductance as a function of piezo voltage measured during the increase of the distance between the electrode apices. The red line is an exponential fit to the tunneling conductance dependence on inter-electrode distance. **Inset**: histogram of the constant K relating the inter-electrode distance and the applied piezo voltage.

Supplementary section 3: Applied voltage effect on the magneto-structural response of Pt wires

Figures S4 and S5 show no apparent effect of applied bias voltage on the reported magneto-structural response, when increasing the bias from 20 mV to 100 mV and 180 mV. This analysis is limited to 180 mV, since we typically observe clear shortening of atomic wires due to Joule heating at above 200 mV or higher bias voltages.

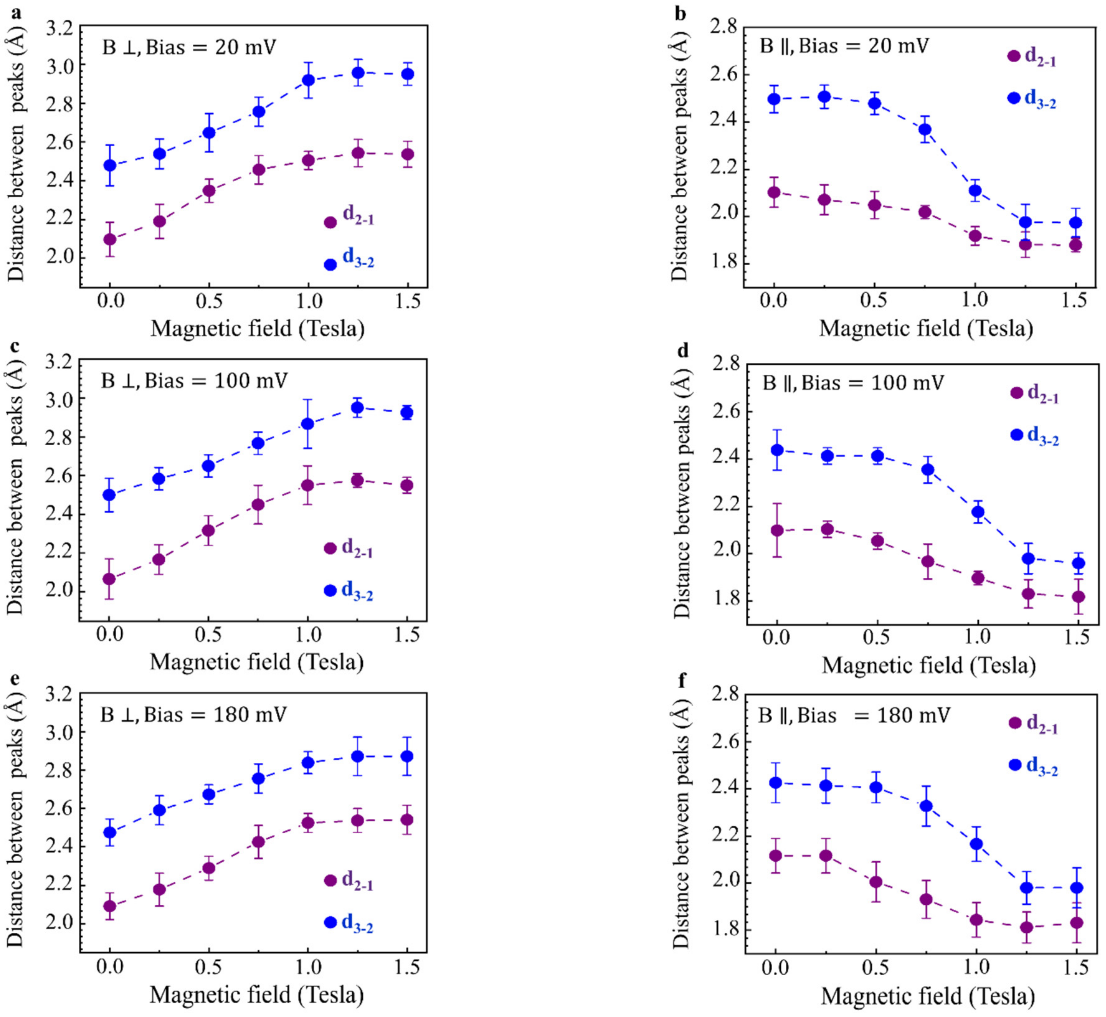

Fig. S4 Magnetic field effect on interatomic distance in Pt atomic wires. a,b, Inter-peak distance as a function of perpendicular (a) and parallel (b) magnetic fields for an applied bias voltage of 20 mV. d_{2-1} and d_{3-2} are the inter-peak distances as defined in Fig. 2 of the main text, which provide an indication for the average interatomic distance in the elongated wires. **c,d,** Similar to a,b, but with an applied bias voltage of 100 mV. **e,f,** Similar to a,b, but with an applied bias voltage of 180 mV. No significant detectable dependence on bias voltage is found. Each data point was obtained from at least 8 length histograms that were collected during different experimental sessions. Each such length histogram is based on 10,000 conductance traces. The error bars provide the standard deviation of the averaged data.

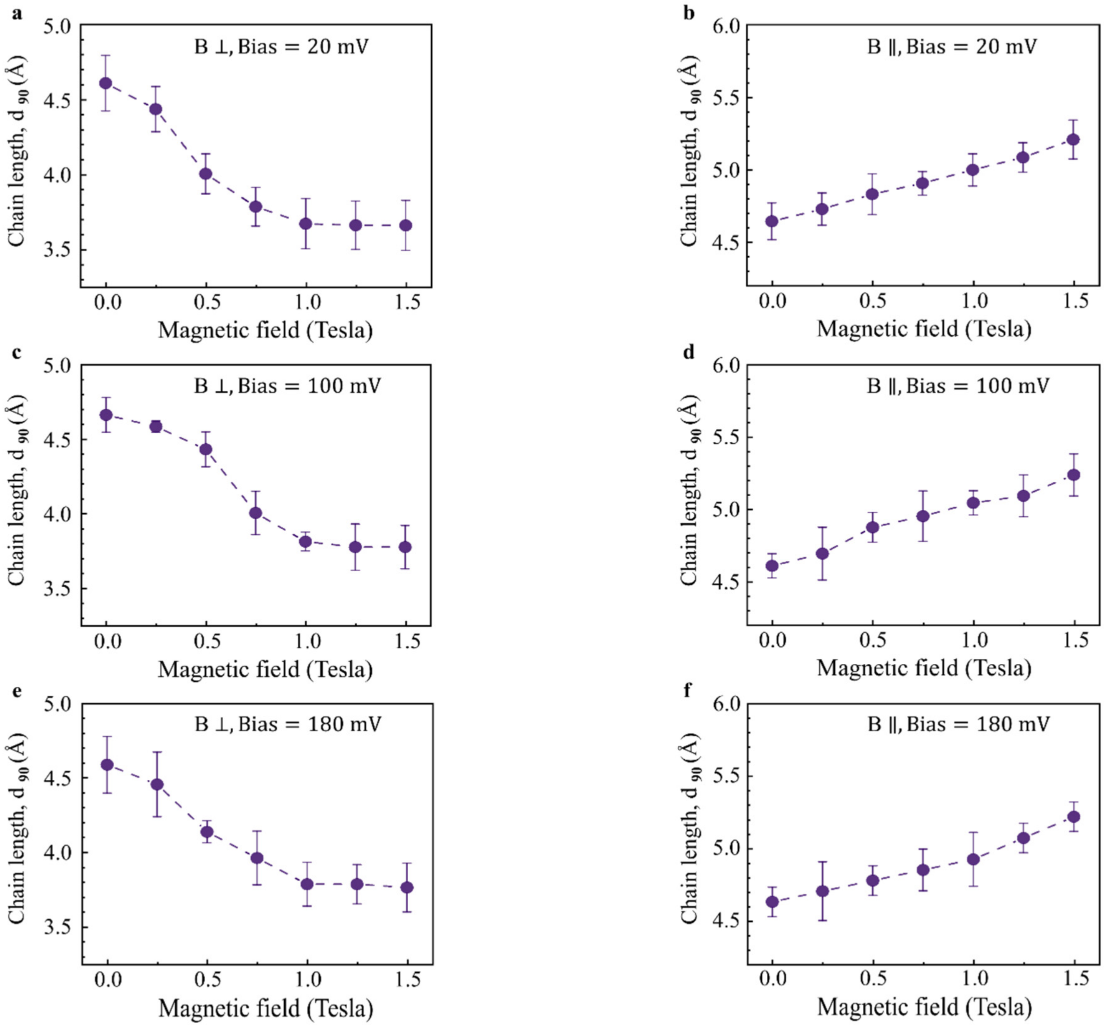

Fig. S5 Magnetic field effect on length of Pt atomic wires. a,b, d_{90}, a measure of the wire length as defined in the main text, as a function of perpendicular (a) and parallel (b) magnetic fields for an applied bias voltage of 20 mV. **c,d,** Similar to a,b, but with an applied bias voltage of 100 mV. **e,f,** Similar to a,b, but with an applied bias voltage of 180 mV. No significant detectable dependence on bias voltage is found. Each data point was obtained from at least 8 length histograms that were collected during different experimental sessions. Each such length histogram is based on 10,000 conductance traces. The error bars provide the standard deviation of the averaged data.

Supplementary section 4: Magneto-structural measurements in Au atomic wires

The magnetization and magnetic anisotropy of Au atomic wires are expected to be negligibe[7]. Therefore, we repeat our entire experimental analysis with Au atomic wires as a control experiment. As can be seen in Fig. S6 and Fig. S7, the inter-peak distance and atomic wire length are not sensitive to parallel or perpendicular magnetic field in the range used in the main text for Pt wires. Interestingly, Fig. S8 shows that the peak width is not affected by a parallel magnetic field, but it increases for a perpendicular magnetic field. This indicates a different magneto-structural effect in comparison to the case of Pt atomic wires. This effect will be reported elsewhere.

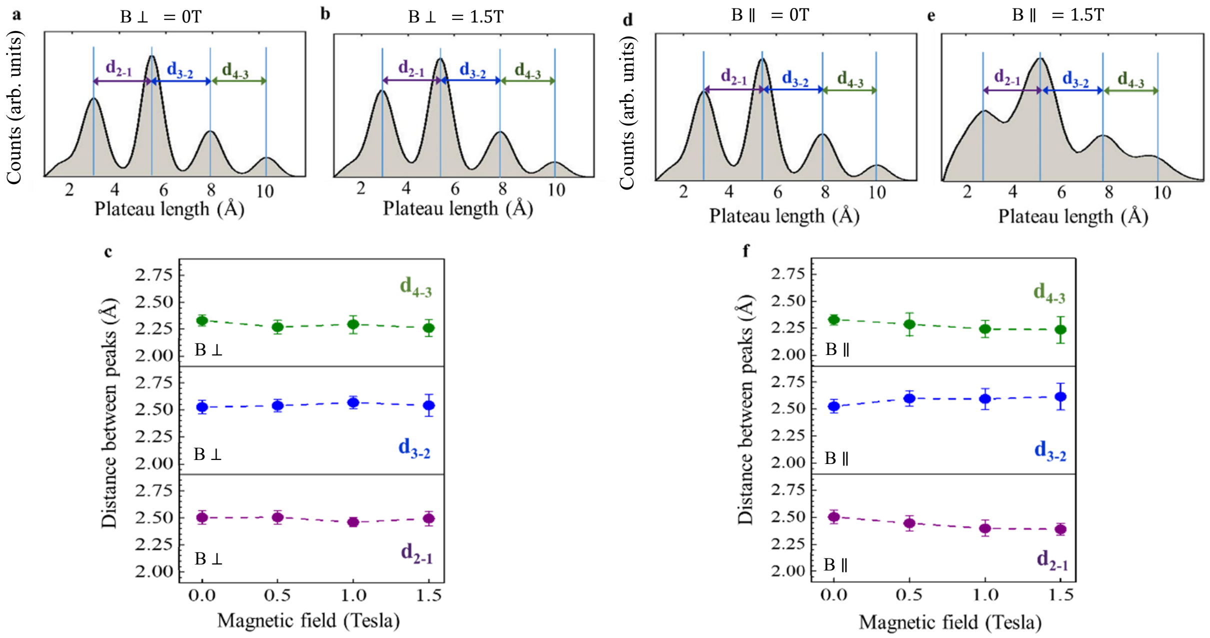

Fig. S6 Inter peak distance in Au length histograms as a function of magnetic field. a,b, Length histograms formed at zero applied magnetic field and at a magnetic field of 1.5 *T* (Tesla) perpendicular to the junction's axis, as defined in Fig. 1a. d_{2-1}, d_{3-2} and d_{4-3} are the inter-peak distances, which are good measures for the average interatomic distance in the elongated wires. **c,** Inter-peak distance as a function of a perpendicular magnetic field. No change in the inter-peak distance is observed within the experimental uncertainty (standard deviation). **d,e,** Similar to a,b but the magnetic field applied parallel to the junction's axis, as defined in Fig. 1a of the main text. **f,** Similar to c but with a parallel magnetic field. Here, as well, no change in the inter-peak distance is observed within the experimental uncertainty. All length histograms are based on 5,000 conductance traces, measured under a bias voltage of 20 mV.

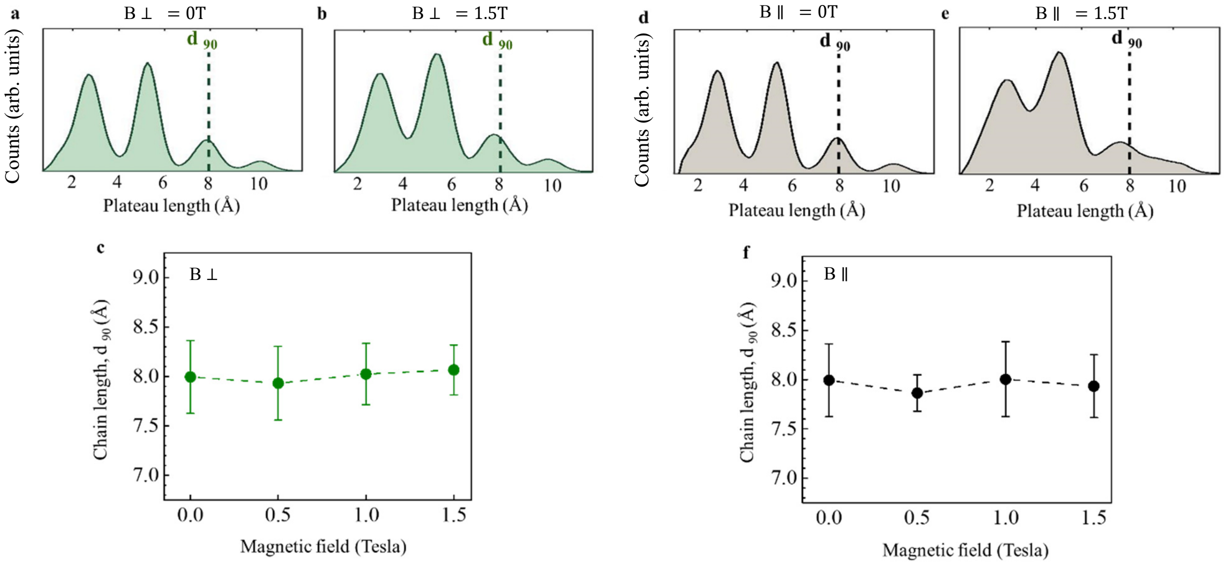

Fig. S7: Wire length for Au atomic wires as a function of magnetic field. a,b, Length histograms formed at zero magnetic field and at a magnetic field of 1.5 *T* applied perpendicular to the junction's axis, as defined in Fig. 1a of the main text. The dashed line represents the location of d_{90}, which is a measure of the wire length as explained in the main text. **c,** d_{90} as a function of a perpendicular magnetic field to the junction's axis. **d,e,** Similar to a,b but with a parallel magnetic field. **f** d_{90} as a function of a parallel magnetic field. Panels c and f compile results of at least 5 measurements. All length histograms are based on 5,000 conductance traces, measured under a bias voltage of 20 mV. The error bars represent the corresponding standard deviation. No change in d_{90} is observed as a function of magnetic field for Au atomic wires within the experimental uncertainty.

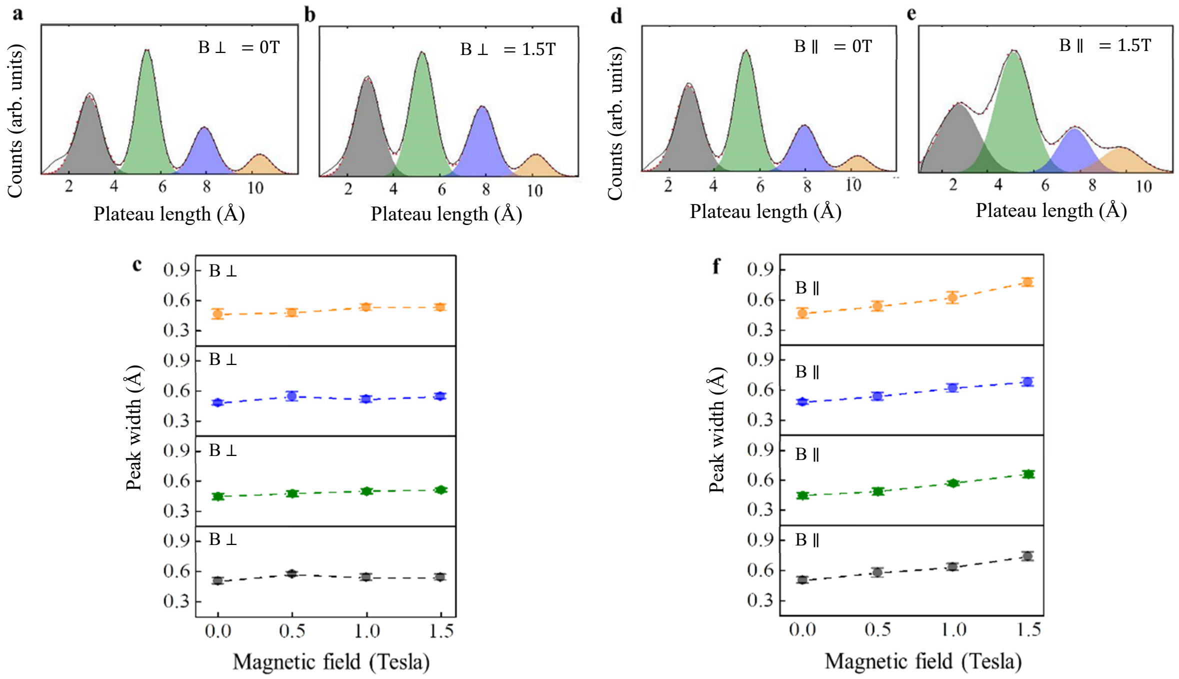

Fig. S8 Peak width for Au atomic wires as function of magnetic field. a,b, Show length histograms for atomic wires in the absence of magnetic field and under a field of 1.5 *T* applied perpendicular to the junction's axis, respectively. The peaks are fitted by Gaussian functions (red dots). **c,** Peak widths (obtained from the Gaussian fitting) as a function of magnetic field strength applied perpendicular to the junction's axis. **d,e,** Similar to a,b, but for a parallel magnetic field. **f,** Similar to c, but for a parallel magnetic field. Panels c,f compile results of at least 5 measurements. The error bars represent the corresponding standard deviation. All length histograms are based on 5,000 conductance traces, measured under a bias voltage of 20 mV.

Supplementary section 5: d_{90} and its response to magnetic field

As mentioned above (and in the main text), to evaluate the length of the Pt atomic wires we have defined the parameter, d_{90}, such that 90% of the formed wires are shorter than its value. This parameter is more reliable than the evaluation of the maximal wire length, which is determined by a limited number of long wires and thus is highly sensitive to variations between different experiments. Fig. S9 presents the location of d_{90} with respect to the corresponding length histograms and the influence of perpendicular and parallel magnetic fields on this parameter. d_{90} reduces in response to a magnetic field applied perpendicular to the junction's axis, whereas it increases when a magnetic field is applied parallel to the junction.

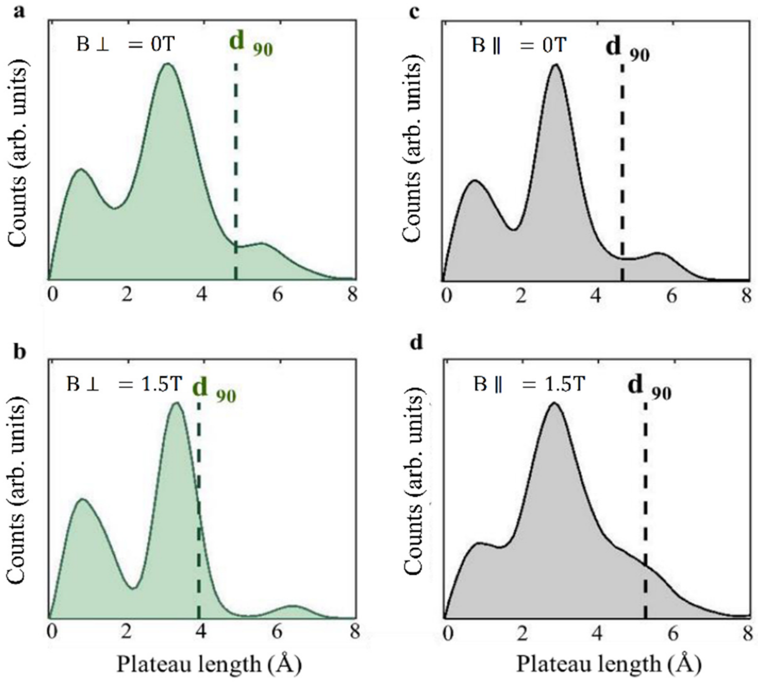

Fig. S9 The location of d_{90} within the Pt length histogram and its response to magnetic field. a,b, Length histograms constructed from 10,000 conductance traces in the absence of magnetic field (a) and in the presence of a magnetic field of 1.5 *T* applied perpendicular to the junction's axis (b). **c,d,** Similar to a,b, but for a parallel magnetic field. The dashed line represents the location of d_{90}. The measurements were performed using a bias voltage of 20 mV.

Supplementary section 6: Comparison between different wire length evaluations

To verify that the observed trends in d_{90} in response to magnetic field direction and strength in Fig. 2 of the main text is not sensitive to the exact definition of the wire length parameter, we examine the magnetic field dependence of d_{70}, and d_{95}. The definition of d_X is analogous to the one of d_{90}, as discussed in Supplementary Section 4 above, with x% of the atomic wires being shorter than d_X. Fig. S10 reveals that the magnitude of the magnetic-induced shifts is different for different d_X, but the shift trends are identical. Namely, the qualitative

behavior is preserved. Similar trends can be seen in Fig. S11 that presents the magnetic-field dependence of the average length of the 10 longest wires.

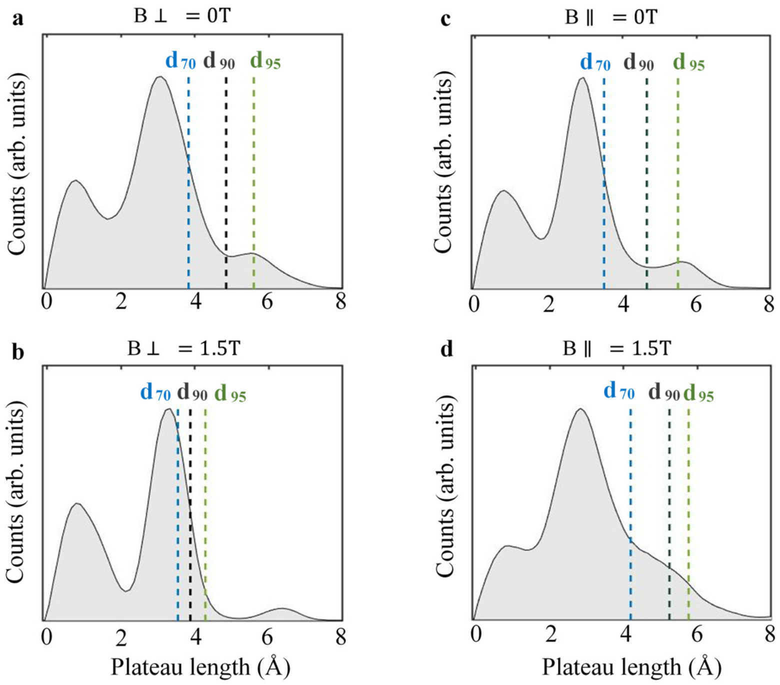

Fig. S10 d_x response to magnetic fields. a,b, Length histograms constructed from 10,000 conductance traces in the absence of a magnetic field (a) and in the presence of a magnetic field of 1.5 *T* applied perpendicular to the junction's axis (b). Vertical dashed lines represent the location of d_x, x=70% (light blue), 90% (black), and 95% (green). **c,d,** Similar to a,b, but for a parallel magnetic field. The measurements were performed using a bias voltage of 20 mV.

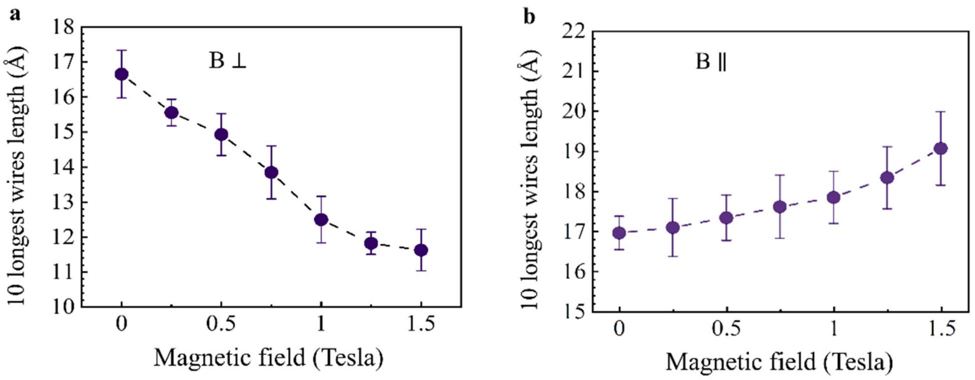

Fig. S11 Longest wires response to magnetic field. Averaged length of the ten longest atomic wire as a function of magnetic field applied perpendicular (a) and parallel (b) to the junction's axis. For each magnetic field, 5 different sets of 10,000 conductance traces each were measured, then the average length of the 10 longest plateaus at ~1.6 G_0 were calculated for each set. The average and standard deviation of these values for the 5 sets are presented.

Supplementary section 7: magnetic field effect on the number of atoms in Pt wires

Figs. S12a,b show an estimation of the average number of atoms in Pt atomic wires as a function of magnetic field. This information is obtained by dividing the wire length by the interatomic distance. Specifically, the wire length is given by d_{90} and the interatomic distance is given by the average inter peak distance ($(d_{3-2} + d_{2-1})/2$) at each magnetic field. Based on Figs. S12a,b, a parallel magnetic field increases the average number of atoms in the wires by 0.7±0.1 atoms and a perpendicular magnetic field decreases it by 0.7±0.1 atoms. We repeat this analysis for the average length of the ten longest atomic wires to get a similar, though pronounced, behavior as seen in Figs. S12c,d. Here, the length increases by 2.3±0.5 atoms or decreases by 3.1±0.4 atoms for the same fields, indicating a higher sensitivity of the longest wires to magnetic fields. This is expected, considering that the wire's magnetization increases for longer wires[8]. This analysis estimates the magnetic field effect on the number of atoms added to or subtracted from the wires, and it does not provide an accurate estimation for the number of atoms in the wires that depends on the exact definition of where the wires begin/end.

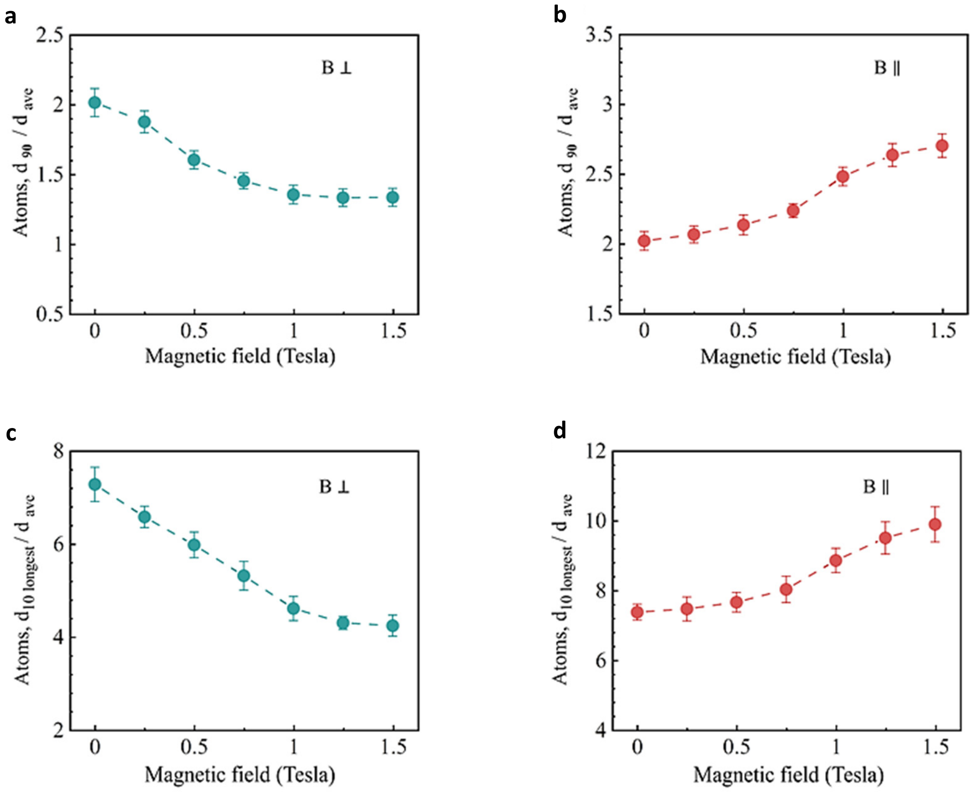

Fig. S12 Number of atoms in Pt atomic wires as a function of magnetic field. a,b, d_{90} divided by the interatomic distance given by the average inter peak distance (d_{ave} =$(d_{3-2} + d_{2-1})/2$), at the relevant magnetic field magnitude. The resulted number of atoms is plotted as a function of perpendicular (a) and parallel (b) magnetic fields. **c,d,** Average length of the longest 10 wires ($d_{10\ longest}$) divided by the average inter peak distance, at the relevant magnetic field magnitude. The resulted number of atoms is plotted as a function of perpendicular (c) and parallel (d) magnetic fields. The number of atoms decreases by 0.68 atoms for the d_{90} analysis (3.07 atoms for the 10 longest wires) when a perpendicular magnetic field is applied, and it increases by 0.69 atoms (2.25 atoms for the 10 longest wires) when a parallel field is applied.

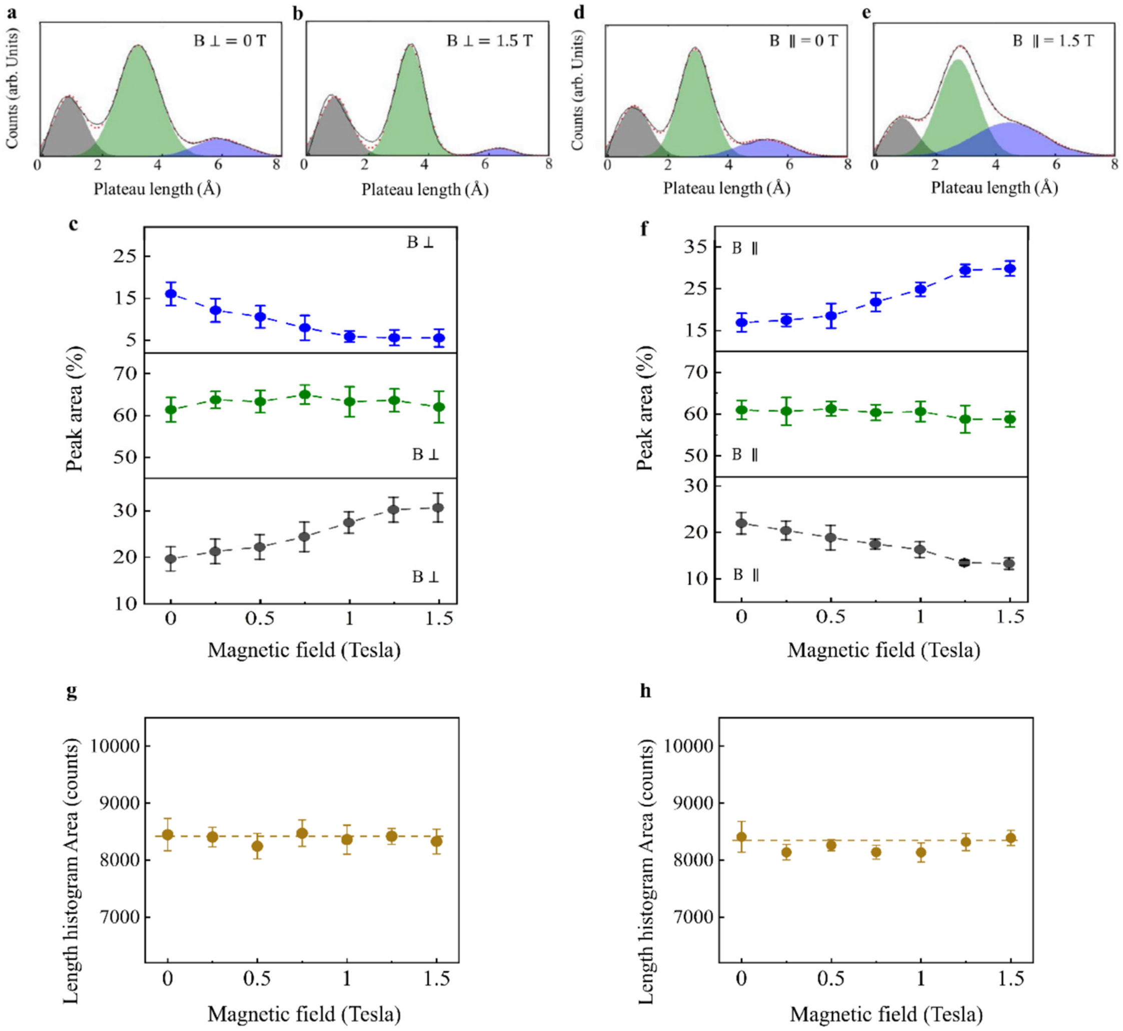

Fig. S13 Magnetic field effect on the peak area in the length histograms of Pt atomic wires. a,b,d,e, Length histograms (based on 10,000 conductance traces measured under a bias voltage of 20 mV) recorded at zero magnetic field (a,d), and at a field strength of 1.5 T applied perpendicular (b) or parallel (e) to the junction's axis. The peaks are fitted with Gaussian functions (red dots) and the sum of these fits appears in black. **c,f,** Areas of the fitted Gaussians as a function of the applied perpendicular (c) and parallel (f) magnetic field strength for the three peaks. The colors correspond to the peak colors appearing in a,b. Each data point provides the average area based on at least 8 length histograms taken at different experimental sessions under the same magnetic field. Error bars represent the corresponding standard deviation. **g,h,** Total areas of length histograms as a function of applied perpendicular (a) and parallel (b) magnetic fields. Each point represents the average area of 8 histograms (c,d) or 5 histograms (g,h). The error bars indicate the variance at each point.

Next, we focus on the relative weights of the peaks in the length histograms. Using Gaussian fittings, Figs. S13a-f present the relative area of each peak with respect to the total area of all peaks as a function of magnetic field. In response to the application of a parallel magnetic field, the area below the 3rd peak increases, while the area bellow the 1st peak decreases. The application of a perpendicular field leads to an opposite trend. Interestingly, the area bellow the 2nd peak is not very sensitive to magnetic fields, indicating that changes in the populations of the 1st and 3rd peaks are done on account of each other, while the population of 2nd peak is not significantly affected. The preservation of the 2nd peak can be understood in the following way. When a parallel field is applied, a larger

number of wires successfully survive rupture at the 1st peak and are being elongated to the 2nd peak. However, a similar number of wires successfully survive rupture at the 2nd peak as well, and are being elongated to the 3rd peak. We note that when examining the total area below the length histogram (Fig. S13g,h), we find a negligible change that can be rationalized as an outcome of the mentioned balance: the pronounced elongation to the 3rd peak is compensated by the suppression of wires that are broken in the 1st peak.

Supplementary section 8: Magnetic field effect on the peak sharpness in the length histograms of Pt atomic wires

In different length histograms taken in the absence of a magnetic field, arbitrary changes in the sharpness of the peaks may take place. This is probably due to changes in the atomic arrangement of the electrode apices. To clearly present the effect of magnetic field on the sharpness (or width) of the peaks in the length histograms, in Fig. 3 of the main text as well as in Figs. S14-S15 (presenting an extended version of Fig. 3), we deliberately choose an initial length histogram with wide (sharp) features at B = 0 *T* and then apply a perpendicular (parallel) magnetic field to exemplify the full range of peak widths response to the magnetic field.

To demonstrate that this choice does not influence our conclusions, in Fig. S16 we start with length histograms of similar sharpness before applying a magnetic field either parallel or perpendicular to the junction's axis. Then, we quantitatively analyze the effect of the magnetic fields on the peak width by fitting Gaussian functions to the peaks and examining the magnetic field influence on the Gaussian widths (Figs. S16a,b,d,e). Figs. S16c,f present the response of the Gaussian width, where each data point is an average based on at least 8 histograms per magnetic field value. For a perpendicular magnetic field, a clear reduction in the peaks' width is seen for the second and third peaks, while there is no clear influence on the first peak. For a parallel magnetic field, an increase in the width of the third peak is observed. The minor increase in the width of the second peak is of the order of the measurement uncertainty and we do not detect any magnetic field influence on the first peak. Former density functional theory (DFT) calculations showed that the magnetic moment in Pt atomic wires increases as a function of their length[8], probably up to saturation. Therefore, shorter atomic wires are expected to have lower or no magnetic moment, explaining the observed reduced sensitivity of such wires to the external magnetic field.

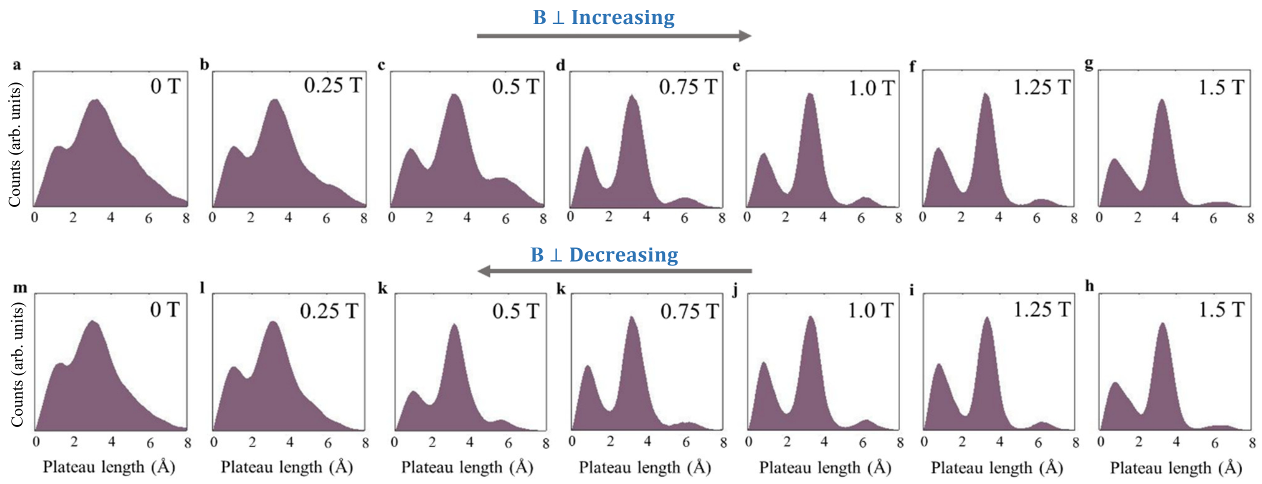

Fig. S14 The effect of perpendicular magnetic field on the peak sharpness in the length histograms of Pt atomic wires. Length histograms recorded sequentially as a function of increasing (a-g) and decreasing (m-h) magnetic field applied perpendicular to the junction's axis. The peaks' sharpness increases for higher magnetic field strengths. This response is inverted when the magnetic field is reduced. All length histograms are based on 10,000 conductance traces measured under a bias voltage of 20 mV.

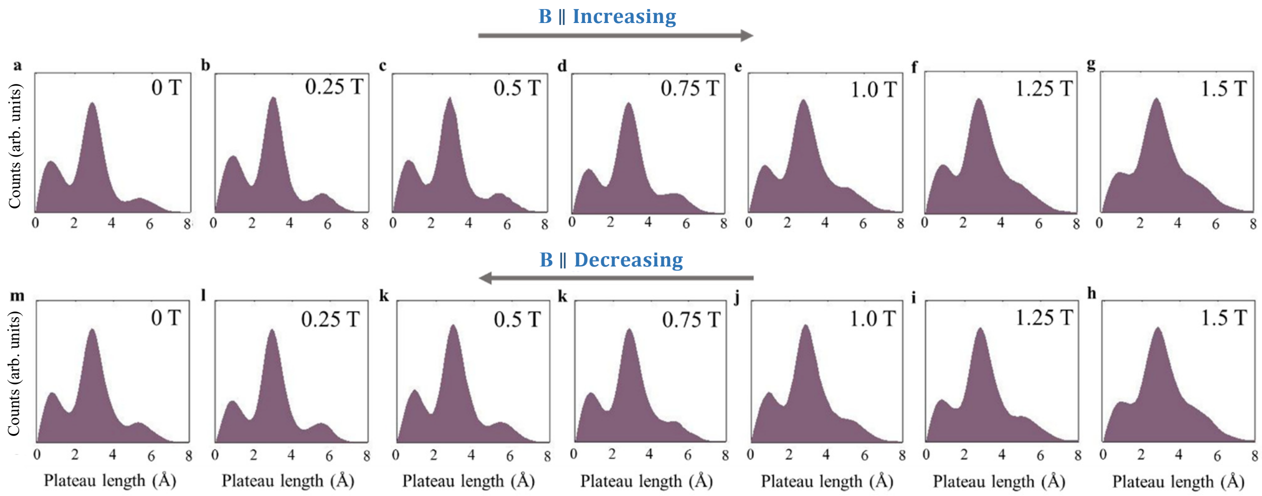

Fig. S15 The effect of parallel magnetic field on the peak sharpness in the length histograms of Pt atomic wires. Length histograms recorded sequentially as a function of increasing (a-g) and decreasing (m-h) magnetic field applied perpendicular to the junction axis. The peaks' sharpness decreases for higher magnetic field strengths. This response is inverted when the magnetic field is reduced. All length histograms are based on 10,000 conductance traces, measured under a bias voltage of 20 mV.

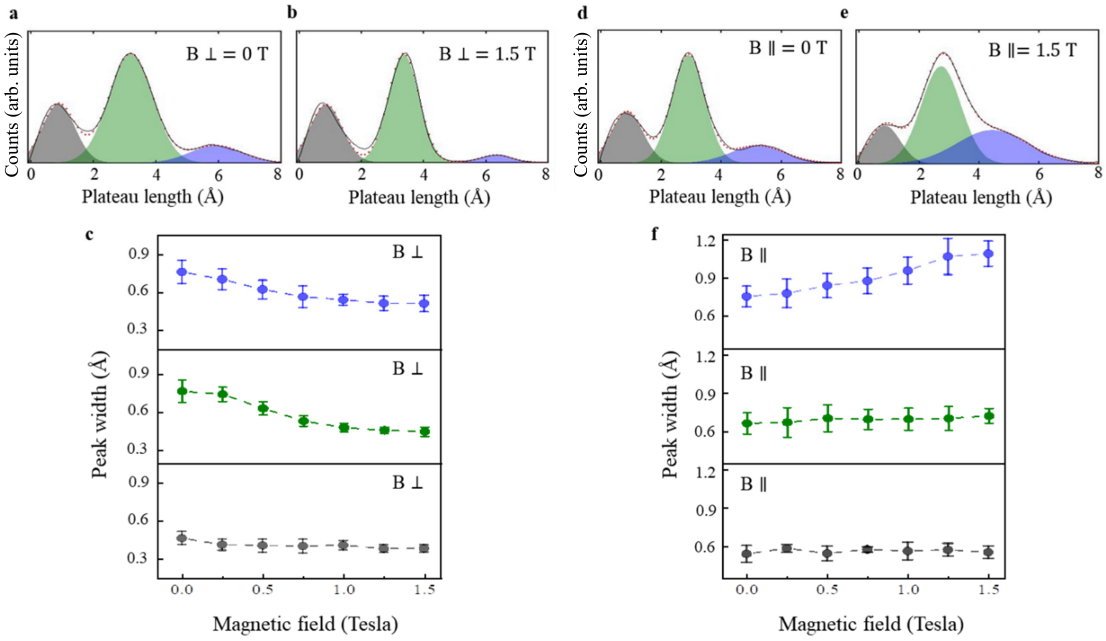

Fig. S16 Magnetic field effect on the peak width in the length histograms of Pt atomic wires. a,b, Length histograms (based on 10,000 conductance traces measured under a bias voltage of 20 mV) recorded at zero magnetic field and at a field strength of 1.5 T applied perpendicular to the junction's axis, respectively. The peaks are fitted with Gaussian functions (red dots) and the sum of these fits appears in black. **c,** Widths of the fitted Gaussians as a function of the applied perpendicular magnetic field strength for the three peaks. The colors correspond to the peak colors appearing in a,b. Each data point provides the average width based on at least 8 length histograms taken at different experimental sessions under the same magnetic field. Error bars represent the corresponding standard deviation. **d,e,** Similar to a,b, but for a parallel magnetic field. **f,** Similar to c, but for a parallel magnetic field.

Supplementary section 9: First-principles calculations

Zigzag to linear atomic wire transition

Pt atomic wires are known to have a zigzag structure near equilibrium[2,3,7]. Nevertheless, upon stretching, the wires become linear[2,3,7]. To obtain a qualitative description of the stretching process from compact zigzag wire configurations up to the breaking point, we performed DFT calculations (see technical details below) on junction models consisting of 1- and 2- Pt atoms bridging two Pt_{14} square pyramidal structures[9], serving as electrode apex models (see Fig. S17). A constrained structural relaxation of the apices and the bridging Pt atoms was performed by keeping the 13 atoms of the base and center layer of each pyramid fixed. The calculation was repeated for several inter-pyramid distances corresponding to junction configurations ranging from compact zigzag to stretched and up to the breaking point of the junction, as seen in Figs. S18a,e. From these calculations, we extracted geometric parameters of the junction (Figs. S18b,c,f,g), including the angles defining the zig-zag structure and the bond lengths within the bridge, as illustrated in Fig. 712. Furthermore, we evaluated the tension developing within the bridge (hereafter termed the force) while pulling the electrodes apart, by forward two-point numerical differentiation of the total energy for a set of relaxed structures of different inter-electrode separations. In this procedure, we first set the inter-electrode separation, then we relax the wire structure while keeping the apex model bases (13 atoms) fixed, and finally we shift both apex bases along the junction's axis to increase the inter-electrodes separation and perform a single-point energy evaluation. The force is evaluated as the numerical derivative of the energy corresponding to the relaxed and stretched configurations with respect to the inter-electrode separation. The resulting force-distance curves appear in Figs. S18d,h. Our calculations show that the Pt atomic wire adopts a linear configuration during stretching (zero zigzag angle) at an early stage before the rupture event (Figs. S18b,f), in agreement with former calculations done for infinite Pt atomic wires[5]. This suggests that a linear wire model is required when studying the wire breaking process.

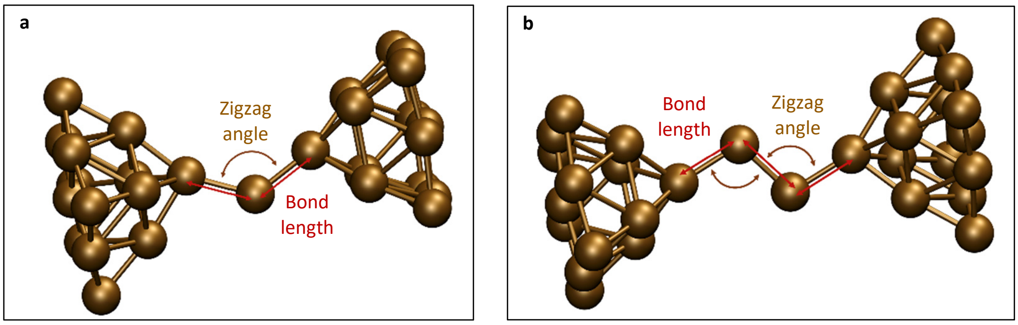

Fig. S17 Schematic representation of 1- and 2- Pt atoms bridging two Pt_{14} square pyramidal model electrode apices. a, Zigzag angle and bond length for the 1-Pt atom junction used in Figs. S18a-d. **b,** Zigzag angles and bond lengths for the 2-Pt atom junction used in Figs. S18e-h.

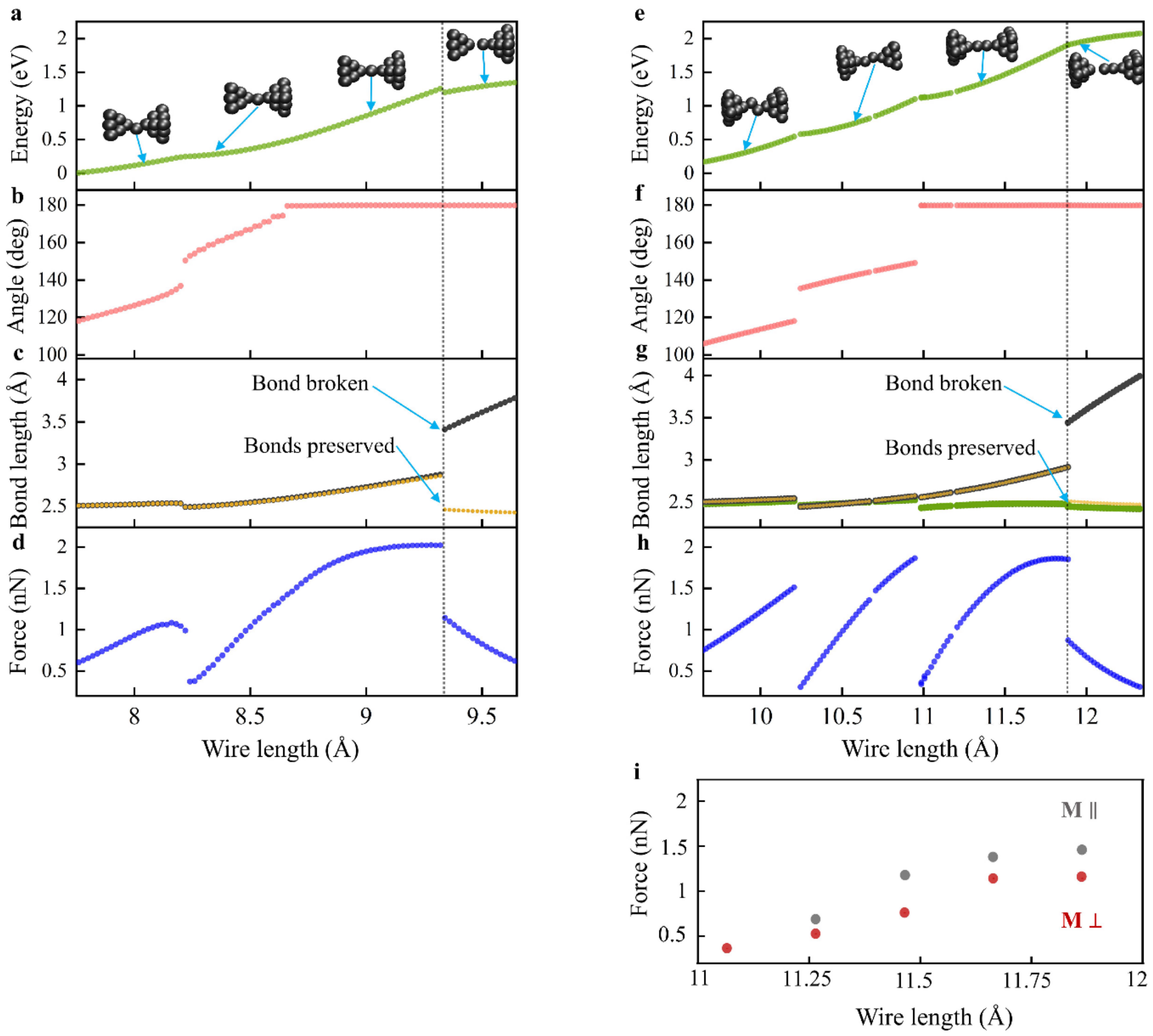

Fig. S18 Zigzag to linear transition and non-collinear spin DFT calculations for Pt atomic wire between electrode apices. Spin-compensated calculations for 1 atom (a-d) and 2 atoms (e-h) bridging the two electrode apex models. **a,e,** Total energy as a function of wire length. **b,f,** Zigzag angle (see Fig. S17) as a function of wire length (inter-electrode separation). In f, the average of the two zigzag angles is presented. **c,g,** Bond length as a function of wire length. An abrupt increase in bond length indicates bond rupture. **d,h,** Force as a function of wire length. The wire is broken at the maximal force. **i,** Non-collinear spin DFT force calculations as a function of wire length for parallel (black) and perpendicular (red) magnetizations (2 atoms bridge is used).

Bare finite atomic wires

Since we are interested in the breaking process, which occurs when the wires are stretched and linear, we next focus on linear wires. In the main text, we have deduced, based on experimental observations, that the force as a function of wire elongation (and hence the total energy curve) depends on the magnetization orientation within the wire. To validate this assumption, we adopted simple, finite linear wire models and performed non-collinear DFT calculations, including spin-orbit interactions, to evaluate the force acting on the wire's edge atoms (prone to rupture, based on our calculations) during wire stretching. This approach extends previous collinear spin

calculations by Thiess *et al.*[10] that compared the elongation tendency of metallic atomic wires in their non-magnetic and (anti)ferromagnetic states. More elaborate non-collinear spin DFT calculations, including explicit electrode apices models are presented below. Since non-collinear spin calculations can lead to multiple solutions with different spin orientations and configurations, we took special care in preparing the starting initial guesses guiding the self-consistent iterations to target solutions with magnetization either perpendicular or parallel with respect to the Pt wire's main axis. We started the non-collinear spin calculations from collinear spin initial guesses with a spin polarization perpendicular and parallel to the wires, for wires of 4-6 equidistant Pt atoms with 3 to 13 unpaired electrons. Once the non-collinear spin calculations converged, we displaced the Pt atoms to a slightly stretched or compressed configuration (with the same number of wire atoms) and performed a new self-consistent calculation using the previous density as a starting guess. To select the solutions that better represent the Pt wire in the presence of electrodes, we discarded solutions with sizable atomic magnetization at the edge atoms[8].

Figure S14a presents the total energy as a function of length for linear wires of different number of atoms, with magnetization parallel or perpendicular to the wire axis. Our results show that the energy of the stretched wires (beyond equilibrium) is sensitive to the magnetization orientation. Focusing on the wire breaking process, we plot in Figs. 4b,c the calculated force on the peripheral wire atoms during stretching. The force curves for parallel and perpendicular magnetization are clearly different. In view of the distinct energy and force curves for the orthogonal magnetizations, one may expect that the interatomic distance at the breaking point and wire stability during elongation is affected by controlling the magnetization direction in the wire.

<u>Non-collinear spin DFT calculations for Pt atomic wires between electrode apices</u>

To examine the effect of electrode apices on our stretched wire calculations, we selected a few 2-atom junction structures, in the stretched bond region prior to the breaking point, from the zigzag to linear atomic wire transition calculations (described above) and performed non-collinear spin DFT calculations including the spin-orbit interaction for spin magnetizations parallel and perpendicular to the wire's axis. Because the electrodes are not semi-infinite as in the experiments, the magnetization may contaminate the small Pt atomic clusters. Therefore, we took special care to obtain solutions that involve minimal spin polarization on the electrode apices. To this end, we started the non-collinear spin calculations from fragment guesses with closed-shell electrode pyramids and spin polarized bridge atoms with spin orientation parallel and perpendicular to the wire's axis. The resulting force-distance curves are shown in Fig. S18i. For a given wire length, a smaller force is obtained for the perpendicular magnetization orientation than for the parallel one, in line with our results for the bare wire model calculations presented in Fig. S19, supporting the assumptions made in the minimal model described below.

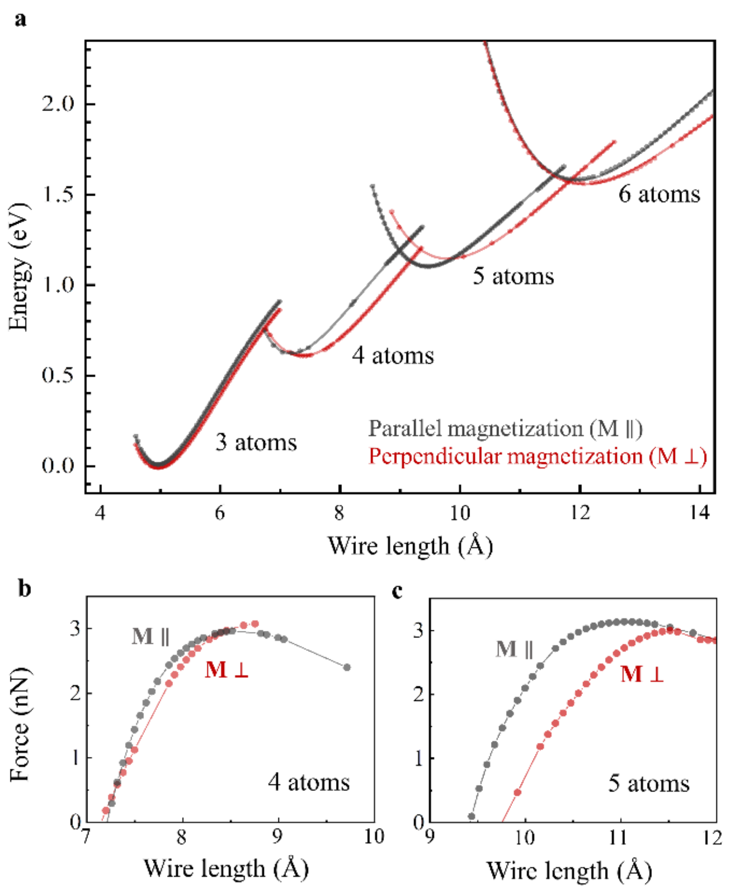

Fig. S19 Non-collinear spin DFT calculations for finite Pt atomic wires. a, Total energy as a function of wire length for wires of 3-6 atoms with parallel and perpendicular magnetization. When the wires are stretched beyond the equilibrium length (at minimum energy), the energy versus length dependence is different for the two magnetization orientations. Since emphasis is put on stretched configurations, linear wires are considered, with equal interatomic distance as a simplification. **b,c,** Force on one of the peripheral atoms as a function of wire length for wires composed of 4 (b), and 5 (c) atoms with parallel and perpendicular magnetizations.

Technical details

All DFT calculations were performed with the Gaussian Suite of programs[11], using the PBEh hybrid density functional approximation[12-14]. This particular DFT approximation, which admixes 25% of Hartree-Fock exchange with 75% of PBE exchange and 100% PBE correlation, was chosen due to three important features: a) it can be derived from first-principles; b) it admixes orbital-dependent Hartree-Fock type exchange, which has been identified as a necessary component to accurately capture spin-orbit effects[15]; and c) it reduces the self-interaction error, which negatively affects density functional calculations involving stretched bonds[16]. The Stuttgart-Cologne energy-consistent relativistic (10 electrons) small-core effective core potential, including the spin-orbit component for the non-collinear spin calculations, was employed along with the corresponding aug-cc-pVDZ-PP basis-set[17]. A self-consistent convergence criteria of maximal variations of 10^{-6} a.u. in the root mean square density matrix and energy between any two consecutive iterations was employed throughout. No explicit

magnetic field was included in the calculations as it has a negligible effect on the calculated total energies and properties, considering the experimentally relevant magnetic field range of $B \leq 1.5\ T$.

Supplementary section 10: Description of the minimal model

A simplified description of the process that takes place in the break-junction experiments presented in this work can be summarized as follows. Initially, two metallic Pt electrodes are pulled apart until a monatomic Pt wire is formed. Upon further pulling apart the electrodes, the wire stretches and can potentially extract another atom from one of the electrodes to form a wire longer by one atom. This will occur provided that the force required for pulling an atom from an electrode does not exceed the force required for wire rupture (maximal force in Fig. 4b of the main text). Providing a fully first-principle description of the wire elongation and rupture processes in the presence of a magnetic field is an intractable problem. Therefore, in order to rationalize the experimental results presented above, we introduce a phenomenological model that captures the main physical aspects of the involved processes. In what follows, we present the essentials of this model.

The basic ingredients and simplifying assumptions of the model are as follows:

1. Out of the intricate manifold of non-collinear spin states that the system has, we consider only two coherent spin states corresponding to magnetization directed in parallel to the wire direction (longitudinal) or perpendicular to it (transverse). This is consistent with the orientation of the external magnetic field relative to the wire axis in the experimental setup. These two extreme magnetization orientations are expected to be the most susceptible to the application of the magnetic field. Other states, such as essentially antiferromagnetic states, are less influenced by the external magnetic field.
2. Since in our experimental setup we focus our attention on wire rupture processes, where the wires are stretched beyond the transition point from zigzag to linear configuration[18], we limit the scope of the rupture model to linear atomic wires, as well. This assumption is supported by the above DFT calculations of the wire's structure during elongation and rupture.
3. Unstretched Pt wires are known to be non-magnetic. Upon stretching, however, magnetism develops[7,18-20].
4. Once magnetization arises, its direction remains predominantly unchanged during the entire stretching process up to the wire elongation point, due to an energetic barrier[20]. See discussion below regarding the height of this barrier.
5. Upon wire elongation, a rapid structural rearrangement occurs, due to the insertion of the additional atom. As a result, the mechanical strain within the wire reduces, leading to near-equilibrium structures and hence suppression of magnetization preference.

6. Wire breaking occurs when the force exerted on its peripheral atoms exceeds the maximal force, which is reached at a different wire length for each magnetization (see Fig. 4b of the main text). This picture is supported by our experimental observations, as well as by ab-initio calculations presented above.

7. In the absence of a magnetic field the wire can adopt either perpendicular or parallel magnetization during stretching. Therefore, when performing many repetitions of the experiment, two distributions of the breaking length will appear for a wire with a given number of atoms, each corresponding to a different magnetization direction. Note that, we assume that in the experiment the separation between the peaks of these two distributions is small, and the distributions of breaking length for a wire with a given number of atoms is seen as a single peak in the experimental length histogram. For the purpose of the present model we assume that these distributions are Gaussian and denote them as $G_t^n(d; d_t^n, \sigma_t) \equiv G_t^n(d)$ and $G_l^n(d; d_l^n, \sigma_l) \equiv G_l^n(d)$ for the perpendicular (transversal) and parallel (longitudinal) magnetization orientations, respectively, where d is the wire length at the breaking point and n is the peak index. Note that to avoid notational confusion between the orientation of the external magnetic field (denoted as perpendicular ($\perp$) or parallel ($\|$)) and the orientation of the spin magnetization of the wire, we use the terms transversal (t) and longitudinal (l) to describe the perpendicular and parallel magnetization orientations, respectively. With this notation, d_t^n and d_l^n are the mean values of the two distributions of breaking lengths, σ_t and σ_l are their respective widths. The latter are chosen to be $\sigma_t = \sigma_l = 0.5$ Å, which reproduces well the experimental peak width in the length histograms (sensitivity tests against this parameter are shown in Supplementary section 11).

8. Once the magnetic field is switched on, the relative weights of these two distributions change, and the probability to form wires with one magnetization orientation increases, while the probability to form wires with the other magnetization orientation decreases, depending on the direction of the external field. This, in turn, causes the observed shift in the combined peaks envelope that are experimentally observed.

Parallel Magnetic Field

To evaluate the relative probability to adopt one of the two considered magnetization orientations as a function of the magnetic field direction and magnitude as well as the temperature, we assign standard Boltzmann weights to each distribution. For the case of a parallel magnetic field applied along the wire direction (chosen as the z direction here), only longitudinal spin configurations aligned parallel (z) and anti-parallel $(-z)$ to the wire axis will be Zeeman shifted by the magnetic field, yielding the following Boltzmann weights:

$$w_{\|}^{z}(B) = e^{\frac{\mu B}{k_B T}},$$

$$w_{\parallel}^{-z}(B) = e^{-\frac{\mu B}{k_B T}},$$

where μ is the effective magnetic moment of the wire (see Supplementary section 9), B is the strength of the magnetic field, and $k_B T$ is the thermal energy. The energetics of the transversal (x and y) spin states remains unaffected by the field resulting in four equivalent spin directions (two, one positive and one negative, along each transversal direction) that are equally populated, giving a total Boltzmann weight of:

$$w_{\parallel}^{x}(B) = w_{\parallel}^{y}(B) = 2e^{0},$$

where the factor of 2 stands for the two degenerate spin orientations. Using these weights, the overall probability distribution for a wire elongation event to occur as a function of parallel magnetic field intensity and wire length can now be expressed as:

$$D_{\parallel}^{n}(d;B) = \left[\frac{w_{\parallel}^{z}(B)+w_{\parallel}^{-z}(B)}{W_{\parallel}(B)}\right] G_{l}^{n}(d) + \left[\frac{w_{\parallel}^{x}(B)+w_{\parallel}^{y}(B)}{W_{\parallel}(B)}\right] G_{t}^{n}(d),$$

where the partition function $W_{\parallel}(B)$ is given by the sum of all unnormalized weights:

$$W_{\parallel}(B) = w_{\parallel}^{z}(B) + w_{\parallel}^{-z}(B) + w_{\parallel}^{x}(B) + w_{\parallel}^{y}(B).$$

The wire $n \rightarrow n+1$ elongation length is then defined as the peak position, $p_{\parallel}^{n}(B)$, of the resulting combined distribution $D_{\parallel}^{n}(d;B)$ for any given magnetic field and temperature. To make direct comparison with the experimental results we then plot the inter-peak distance for the parallel field case:

$$d_{(n+1)-n} = p_{\parallel}^{n+1}(B) - p_{\parallel}^{n}(B).$$

<u>Perpendicular Magnetic Field</u>

Similarly, when the magnetic field points in a direction perpendicular to the wire axis (chosen as the x direction) we have:

$$w_{\perp}^{x}(B) = e^{\frac{\mu B}{kT}},$$

$$w_{\perp}^{-x}(B) = e^{-\frac{\mu B}{kT}},$$

$$w_{\perp}^{y}(B) = w_{\perp}^{z}(B) = 2e^{0},$$

Then, the overall probability distribution for a wire elongation event to occur as a function of magnetic field and wire length can now be expressed as:

$$D_{\perp}^{n}(d;B) = \left[\frac{w_{\perp}^{z}(B)}{W_{\perp}(B)}\right] G_{l}^{n}(d) + \left[\frac{w_{\perp}^{x}(B)+w_{\perp}^{-x}(B)+w_{\perp}^{y}(B)}{W_{\perp}(B)}\right] G_{t}^{n}(d),$$

with the partition function:

$$W_{\perp}(B) = w_{\perp}^{x}(B) + w_{\perp}^{-x}(B) + w_{\perp}^{y}(B) + w_{\perp}^{z}(B).$$

The wire $n \rightarrow n+1$ elongation length is then defined as the peak position, $p_{\perp}^{n}(B)$, of the resulting combined distribution $D_{\perp}^{n}(d;B)$ for any given magnetic field and temperature. To make direct comparison with the experimental results we then plot we then plot the inter-peak distance for the perpendicular field case:

$$d_{(n+1)-n} = p_{\perp}^{n+1}(B) - p_{\perp}^{n}(B)\ .$$

The energy barrier height between parallel and perpendicular wire magnetizations

In the model description, presented earlier in this section, an assumption was made that following elongation, when an atom is inserted into the wire, the wire's strain is partially relaxed and the magnetization preference is suppressed[7,18-20], thus facilitating magnetization alignment by a relatively small magnetic field. As the elongated wire is further stretched, parallel and perpendicular magnetization states develop with a corresponding energy barrier separating them (see point number 4 above). To support this picture and demonstrate that the barrier height is larger than the thermal and Zeeman energies under the experimental conditions, we revisit the results of the temperature-dependent experiments appearing in Figs. 4i,j of the main text. Two important observations can be drawn: (i) as discussed in the main text and predicted by our minimal model, the effect of varying the temperature is translated to a change in the saturation magnetic fields (dashed red line in Figs. 4i,j) due to the Boltzmann competition between the thermal and Zeeman energies; and (ii) at any of the three temperatures considered, we find an identical saturation magnetic field for the parallel and perpendicular field orientations. The later observation indicates that the energetic barrier between the two magnetization configurations is larger than the thermal energy in the experiment. To understand this, let us assume that the magnetization barrier is lower than or comparable to the thermal energy. In such a case, starting from the suppressed magnetization state of the partially relaxed elongated wire, the application of a parallel magnetic field favors the parallel magnetization state over its perpendicular counterpart. Elongating the wire will not lead to magnetization locking, due to the low barrier, and thermodynamic considerations will lead to thermal population favoring the lower energy perpendicular magnetization state. To reach saturation with a parallel magnetic field, would then require a very high field strength, beyond the value used in our experiments, that would lower the energy of the parallel magnetization state below that of its perpendicular counterpart. In contrast, to reach saturation with a perpendicular magnetic field, one would need to apply a much lower field strength since the population of the perpendicular magnetization state would be thermodynamically dominating already in the absence of a magnetic field. This would be translated to different saturation field values for the two field orientations at any given

temperature, in contrast to our experimental observations. The fact, that we observe the same parallel and perpendicular saturation fields for the three different temperatures considered, indicates that the barrier height is considerably larger than experimental thermal energy, such that at the experimental timescale there is no thermal drift of population from the less stable longitudinal magnetization state to the energetically favorable transverse magnetization state of the stretched wires. We further note that the Zeeman energy in our experiments is of the order of the thermal energy and therefore also smaller than the barrier height of the stretched wire under the experimental conditions. This, therefore, makes it impossible to affect the wire's magnetization state using magnetic fields, once the wire is stretched.

Supplementary section 11: Model sensitivity with respect to input parameters

Our model includes three free parameters, the elongation distribution width (σ), the magnetic moment of the Pt wire (μ), and the breaking length (d). These parameters are defined in Supplementary section 10. To test the sensitivity of our model outcomes with respect to its input parameters, we repeated the calculations presented in the main text for several values of the distribution width, effective magnetic moment, and the breaking length. As shown below, the qualitative nature of our results is insensitive to the value of these parameters within reasonable physical bounds.

<u>Distribution Width σ</u>

The choice of the widths σ_t and σ_l of the distribution functions $G_t^n(d)$ and $G_l^n(d)$ affects the resulting distributions $D_{\parallel}^n(d;B)$ and $D_{\perp}^n(d;B)$ and thus also the calculated peak positions, $p_{\parallel}^n(B)$ and $p_{\perp}^n(B)$. These widths are used as empirical fitting parameters to obtain good agreement with the experimentally measured peak widths as appearing in the length histograms. In the main text, we presented results obtained using a value of $\sigma_t = \sigma_l = 0.5$ Å. To evaluated the robustness of this choice we repeated the calculations of the inter-peak distance dependence on the magnetic field, using slightly smaller and larger values of $\sigma_t = \sigma_l = 0.4$ Å and $\sigma_t = \sigma_l = 0.6$ Å, respectively. As can be seen in Fig. S20, the results are weakly sensitive to variations of the width parameters within the range considered, regardless of the magnetization orientation.

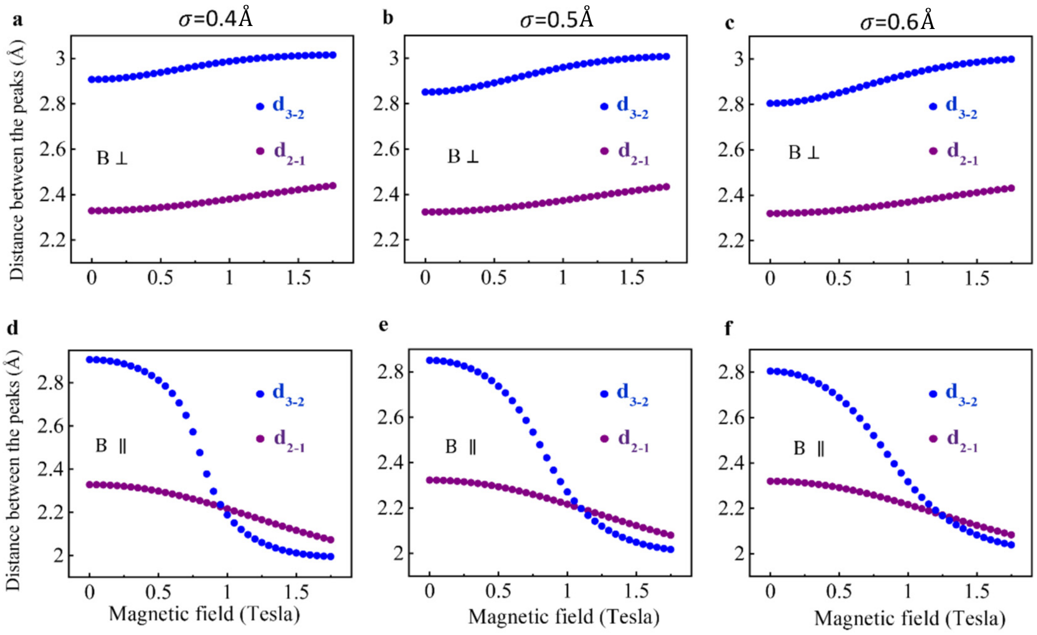

Fig. S20 Model sensitivity test against the peak width parameter. Calculated inter-peak distances as a function of perpendicular (upper panels) and parallel (lower panels) applied magnetic field for $\sigma_t = \sigma_l = 0.4$ Å (panels a and d), 0.5 Å (panels b and e), and 0.6 Å (panels c and f). The calculations are performed using $\mu_0 = 2.5\ \mu_B$.

Effective Magnetic Moment μ

The effective magnetic moment that enters in the weight functions discussed above, can be written as $\mu = n\ \mu_0$, where n is the number of Pt atoms in the wire and μ_0 is the average magnetic moment per atom. This allow us to use the same μ_0 for the different wires as an additional simplification. Comparing the magnetic field dependence of the inter-peak positions obtained from our model with the experimental results, one can estimate the value of $\mu_0 = 2.5\ \mu_B$, used to obtain the results presented in the main text. As reference, the magnetic moment of a stretched Pt wire was reported in the literature by several authors. Fernández-Seivane *et al.*[7] predicted a spin magnetic moment of approximately 1.25 μ_B per Pt atom, using generalized gradient DFT approximation (GGA) calculations on stretched linear and zig-zag wires. Smogunov *et al.*[20] estimated a total magnetic moment per Pt atom (with comparable spin and orbital contributions) of approximately 1.5 μ_B, using the GGA. Considering that GGA tends to underestimate the calculated magnetic moments[21], we believe that our estimated value is within reasonable physical bounds. Nonetheless, to assess the robustness of our predictions towards variations in the value of μ_0, we repeated the calculations of the inter-peak distance dependence on the magnetic field using slightly smaller and larger values of $\mu_0 = 2.0\ \mu_B$ and $3.0\ \mu_B$, respectively. As can be seen in Fig. S21, the general trend is not affected by varying the effective magnetic moment within the considered range, for both magnetization orientations.

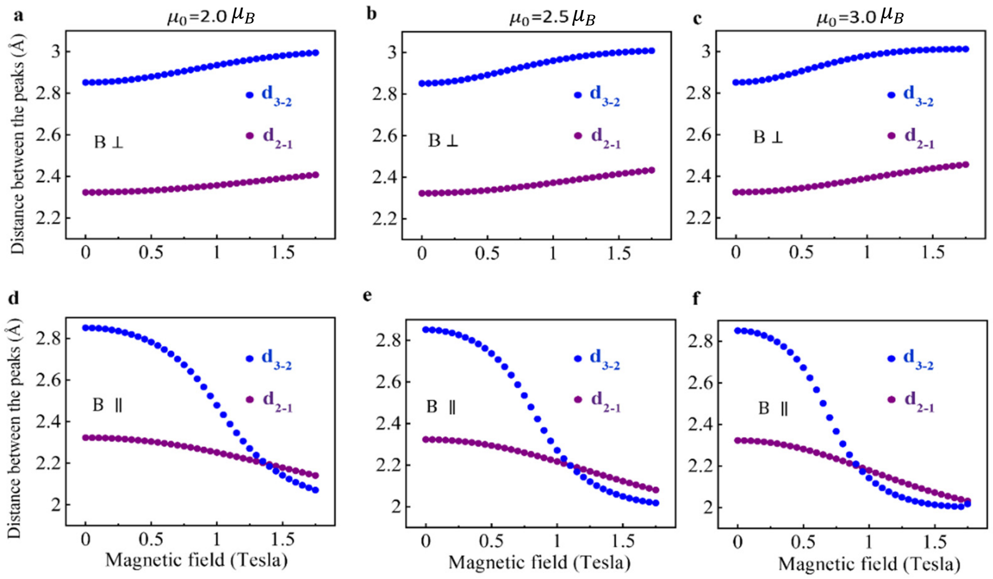

Fig. S21 Model sensitivity test against the effective magnetic moment parameter. Calculated inter-peak distances as a function of applied perpendicular (upper panels) and parallel (lower panels) magnetic field for $\mu_0 = 2.0\ \mu_B$ (panels a and d), 2.5 μ_B (panels b and e), and 3.0 μ_B (panels c and f). The calculations are performed using $\sigma = 0.5$ Å.

Breaking length

For a wire with a given number of atoms, the parallel and perpendicular breaking lengths ($d_{\parallel}$, $d_{\perp}$) are defined as the stretching lengths at which the force is maximal (see Fig. 4b of the main text). These breaking lengths can be extracted from the experiments by considering the peak positions at saturation ($B >$1.25 *T* for Pt atomic wires). In these conditions and at zero temperature, the positions of the peaks provide the breaking length of wires with magnetization along the applied magnetic field. However, at the experimental temperature (5.1 K) this method underestimates the experimental breaking lengths by roughly 20% due to the thermal energy competition with the Zeeman energy. To check the sensitivity of the model results with respect to the values of the breaking lengths, we present in Fig. S22 the calculated inter-peak distance dependence on the magnetic field strength using breaking lengths increased by 10% and 20%, with respect to that used in Fig. 4. The overall quantitative behavior is found to be only mildly sensitive to the value of the breaking length with no influence on the qualitative nature of the results.

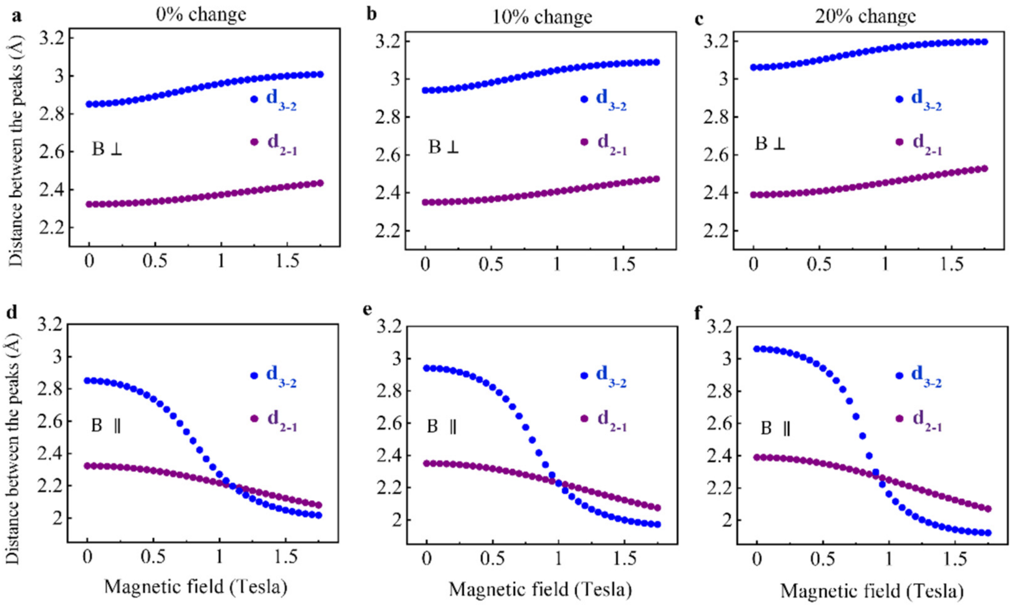

Fig. S22 Model sensitivity test against the breaking length. Calculated inter-peak distances as a function of applied perpendicular (upper panels) and parallel (lower panels) magnetic field for the obtained breaking length based on the saturation location of the peaks (a,d), and larger breaking length values by 10% (b,e) and 20% (c,f). The calculations are performed using $\sigma = 0.5$ Å and $\mu_0 = 2.5\ \mu_B$.

Supplementary section 12: Temperature dependent measurements

The effect of magnetic field on the inter peak distance measured at different temperatures is presented in Fig. S23.

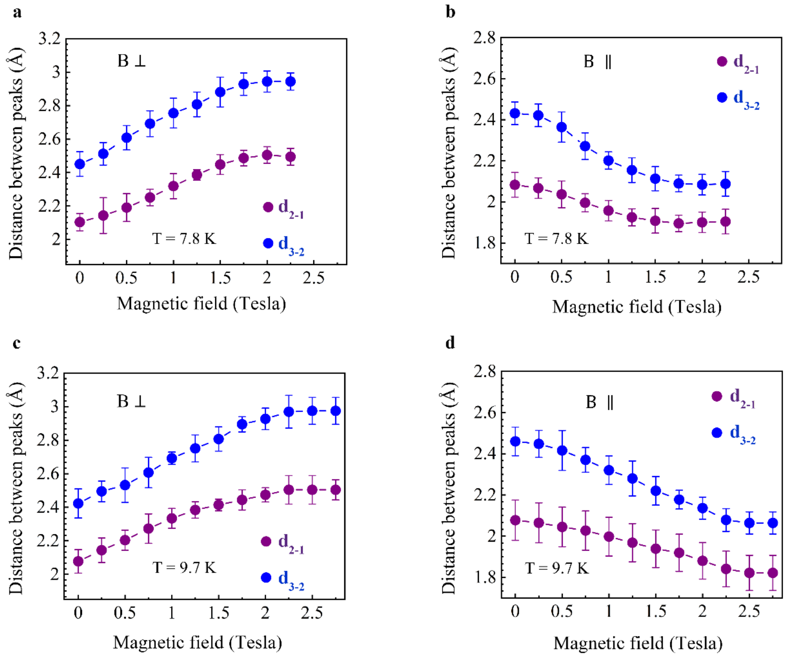

Fig. S23 Magnetic-field-induced structural variations in Pt atomic wires. Inter-peak distance as a function of perpendicular (a,c) and parallel (b,d) magnetic fields at 7.8 K (a,b) and 9.7 K (c,d).

Supplementary section 13: Absence of hysteresis

In contrast to magnetostriction measurements, in our experiments no hysteresis is expected because of the following: (i) An insignificant magnetization preference whenever an atom is inserted to the wire and the interatomic distance is partially relaxed[7,18-20], as supported by the relatively low involved magnetic fields (∼ 1 *T*). (ii) Lack of memory between different wire elongation events. Namely, we compare independent wires that are formed under different magnetic field orientations. Before the formation and characterization of any given wire, we crash the two electrodes into each other to form a contact of about 70-100 atoms to allow the independent formation of a new wire without being affected by past induced magnetic fields and the structure of former wires.

References

1. Strigl, F., Espy, C., Bückle, M., Scheer, E. & Pietsch, T. Emerging magnetic order in platinum atomic contacts and chains. *Nat. commun.* **6**, 1-9 (2015)

2. García-Suárez, V. M., Rocha, A. R., Bailey, S. W., Lambert, C. J., Sanvito, S. & Ferrer, J. Conductance oscillations in zigzag platinum chains. *Phys. Rev. Lett.* 95, 256804 (2005)

3. Vardimon, R., Yelin, T., Klionsky, M., Sarkar, S., Biller, A., Kronik, L. & Tal, O. Probing the orbital origin of conductance oscillations in atomic chains. *Nano Lett.* **14**, 2988-2993 (2014)

42. Yelin, T., Vardimon, T., NKuritz, N., Korytár, R., Bagrets, A., Evers, F., Kronik, L., & Tal, O., Atomically Wired Molecular Junctions: Connecting a Single Organic Molecule by Chains of Metal Atoms, Nano Lett. 13, 1956–1961 (2013)

5. R. H. M. Smit, C. Untiedt, A. I. Yanson, and J. M. van Ruitenbeek, Common Origin for Surface Reconstruction and the Formation of Chains of Metal Atoms, Phys. Rev. Lett. 87, 266102 (2001)

6. R. H. M. Smit, C. Untiedt, G. Rubio-Bollinger, R. C. Segers, and J. M. van Ruitenbeek, Observation of a Parity Oscillation in the Conductance of Atomic Wires, Phys. Rev. Lett. 91, 076805 (2003)

7. Fernández-Seivane, L., García-Suárez, V. M. & Ferrer, J. Predictions for the formation of atomic chains in mechanically controllable break-junction experiments. *Phys. Rev. B* **75**, 075415 (2007)

8. Smogunov, A., Dal Corso, A. & Tosatti, E. Magnetic phenomena, spin-orbit effects, and Landauer conductance in Pt nanowire contacts: Density-functional theory calculations. *Phys. Rev. B* **78**, 014423 (2008)

9. Wei G. F. & Liu Z. P. Subnano Pt Particles from a First-Principles Stochastic Surface Walking Global Search, J. Chem. Theory and Comput. **12**, 4698–4706 (2016)

10. Thiess, A., Mokrousov, Y., Heinze, S. & Blügel, S. Magnetically hindered chain formation in transition-metal break junctions. *Phys. Rev. Lett.* **103**, 217201 (2009)

11. Gaussian 16: Gaussian 16, Revision B.01, Frisch, M. J., *et al.*, Gaussian, Inc., Wallingford CT (2016)

12. Perdew, J. P., Ernzerhof, M. & Burke, K. Rationale for Mixing Exact Exchange with Density Functional Approximations. *J. Chem. Phys.* **105**, 9982-9985 (1996)

13. Ernzerhof, M. & Scuseria, G. E. Assessment of the Perdew-Burke-Ernzerhof Exchange-Correlation Functional *J. Chem. Phys.* **110**, 5029-5036 (1999)

14. Adamo, C. & Barone, V. Toward Reliable Density Functional Methods without Adjustable Parameters: The PBE0 Model. *J. Chem. Phys.* **110**, 6158-6170 (1999)

15. Desmarais, J. K,, Flament, J. P. & Erba, A. Fundamental Role of Fock Exchange in Relativistic Density Functional Theory. *J. Phys. Chem. Lett.* **10**, 3580-3585 (2019)

16. Shahi, C., Bhattarai, P., Wagle, K., Santra, B., Schwalbe, S., Hahn, T., Kortus, J., Jackson, K. A., Peralta, J. E., Trepte, K. & Lehtola, S. Stretched or noded orbital densities and self-interaction correction in density functional theory. *J. Chem. Phys.* **150**, 174102 (2019)

17. Figgen, D., Peterson, K. A., Dolg, M. & Stoll H. Energy-consistent pseudopotentials and correlation consistent basis sets for the 5d elements Hf-Pt. *J. Chem. Phys.* **130**, 164108 (2009)

18. Tung, J. C. & Guo, G. Y. Magnetic moment and magnetic anisotropy of linear and zigzag 4 d and 5 d transition metal nanowires: First-principles calculations. *Phys. Rev. B* **81**, 094422 (2010)

19. Delin, A. & Tosatti, E. Magnetic phenomena in 5 d transition metal nanowires. *Phys. Rev. B* **68**, 144434 (2003)

20. Smogunov, A., Dal Corso, A., Delin, A., Weht, R. & Tosatti, E. Colossal magnetic anisotropy of monatomic free and deposited platinum nanowires. *Nat. Nanotechnol.* **3**, 22-25 (2008)

21. Fernández-Rossier, J., Jacob, D., Untiedt, C. & Palacios, J. J. Transport in magnetically ordered Pt nanocontacts. *Phys. Rev. B* **72**, 224418 (2005)
